# A retrotransposon storm marks clinical phenoconversion to late-onset Alzheimer’s disease

**DOI:** 10.1007/s11357-022-00580-w

**Published:** 2022-05-19

**Authors:** Fabio Macciardi, Maria Giulia Bacalini, Ricardo Miramontes, Alessio Boattini, Cristian Taccioli, Giorgia Modenini, Rond Malhas, Laura Anderlucci, Yuriy Gusev, Thomas J. Gross, Robert M. Padilla, Massimo S. Fiandaca, Elizabeth Head, Guia Guffanti, Howard J. Federoff, Mark Mapstone

**Affiliations:** 1 Department of Psychiatry and Human Behavior, UCI, Irvine, USA; 2 IRCCS Istituto Delle Scienze Neurologiche Di Bologna, Bologna, Italy; 3 Department of Neurology, UCI, Irvine, USA; 4 BiGeA Department, University of Bologna, Bologna, Italy; 5 MAPS Department, University of Padova, Padua, Italy; 6 Department of Statistical Sciences “Paolo Fortunati”, University of Bologna, Bologna, Italy; 7 Georgetown University Medical Center, Washington, DC USA; 8 Department of Pathology, UCI, Irvine, USA; 9 Department of Psychiatry, McLean Hospital, Harvard Medical School, Boston, USA

**Keywords:** Alzheimer disease, Transposable elements, Retrotransposons, Blood biomarkers, Gene expression, Machine learning

## Abstract

Recent reports have suggested that the reactivation of otherwise transcriptionally silent transposable elements (TEs) might induce brain degeneration, either by dysregulating the expression of genes and pathways implicated in cognitive decline and dementia or through the induction of immune-mediated neuroinflammation resulting in the elimination of neural and glial cells. In the work we present here, we test the hypothesis that differentially expressed TEs in blood could be used as biomarkers of cognitive decline and development of AD. To this aim, we used a sample of aging subjects (age > 70) that developed late-onset Alzheimer’s disease (LOAD) over a relatively short period of time (12–48 months), for which blood was available before and after their *pheno*conversion, and a group of cognitive stable subjects as controls. We applied our developed and validated customized pipeline that allows the identification, characterization, and quantification of the differentially expressed (DE) TEs before and after the onset of manifest LOAD, through analyses of RNA-Seq data. We compared the level of DE TEs within more than 600,000 TE-mapping RNA transcripts from 25 individuals, whose specimens we obtained before and after their *phenotypic* conversion (*pheno*conversion) to LOAD, and discovered that 1790 TE transcripts showed significant expression differences between these two timepoints (logFC ± 1.5, logCMP > 5.3, nominal *p* value < 0.01). These DE transcripts mapped both over- and under-expressed TE elements. Occurring before the clinical *pheno*conversion, this TE storm features significant increases in DE transcripts of LINEs, LTRs, and SVAs, while those for SINEs are significantly depleted. These dysregulations end with signs of manifest LOAD. This set of highly DE transcripts generates a TE transcriptional profile that accurately discriminates the before and after *pheno*conversion states of these subjects. Our findings suggest that a storm of DE TEs occurs before *pheno*conversion from normal cognition to manifest LOAD in risk individuals compared to controls, and may provide useful blood-based biomarkers for heralding such a clinical transition, also suggesting that TEs can indeed participate in the complex process of neurodegeneration.

**Supplementary Information:**

The online version contains supplementary material available at 10.1007/s11357-022-00580-w.

## Introduction

Recent findings in genetics are expanding our knowledge of the molecular mechanisms involved in neural cellular aging and neurodegenerative disorders. Some of these mechanisms are not dependent on single gene effects, instead implicating multigenomic and epistatic regulatory features in neurodegeneration (for a recent review, see [1]). In particular, some authors have suggested that the reactivation of otherwise silenced transposable elements (TEs) can impact the neural homeostasis during pathological aging. In these very initial reports, authors hint that de-silenced TEs can either dysregulate the expression of genes and pathways implicated in cognitive decline and dementia or induce neuroinflammatory processes that activate an immune response, and ultimately eliminate neurons and glial cells [2–6].

TEs are a key component of non-coding, regulatory DNA, which makes up 95–98% of the human genome [7–9], and have proven essential for the development and functional organization of the brain through the activation of epigenetic mechanisms [10–12]. Epigenetics helps regulate the neural genome via complex, cooperative molecular mechanisms, including DNA methylation, histone modifications, and non-coding RNA-mediated modulation. TEs are representing by far the largest group of the non-coding RNAs (ncRNAs), yet they remain the least characterized and understood fraction.

Originally, TEs were investigated for their capacity to insert new copies of their sequence into the genome through either direct DNA transposition or RNA-mediated retrotransposition [13–15]. TEs have been considered “selfish” elements, whose only adverse role was to contribute to rare human diseases [16–18]. Over time, their essential genomic regulatory role with a pivotal transpositional activity of TEs in the initial phases of neurogenesis has been appreciated, as well as having important roles in human evolution [11, 19–23]. The majority of human TEs, however, are transpositionally silent in adulthood, because cells developed mechanisms to control TE transposition through methylation, chromatin condensation, and post-transcriptional TE silencing via small RNA, small-interfering RNAs (siRNAs), or PIWI/Argonaute-interacting RNAs (piRNAs) (for a recent review, see [21]). Yet, the limited number of TEs that are still able to escape such silencing machinery has gained considerable interest, as TEs are hypothesized to contribute to genetic variation during development and in somatic tissue differentiation [24–27].

More recently, increased evidence suggests that TEs have evolved to implement epigenetic regulatory functions within the genome, independently from their transpositional activities. As DNA elements, TEs can regulate gene transcription via chromatin modification and by acting as alternative promoters or enhancers [26, 28–37]. When TEs are actively transcribed as ncRNA elements, they can still function to regulate gene expression as expressed promoters or enhancers [22, 30, 38–42], and specifically as enhancer RNAs [43–48], but they can also create new isoforms of protein-coding genes, and post-transcriptionally modify mRNAs, diversifying the proteome [49–52]. Despite many details regarding the TE-dependent regulatory mechanisms that remain inadequately defined, TEs are currently considered key players within the large group of ncRNAs that regulate the expression of protein-coding genes with tissue-dependent and time-dependent functional dynamics [37, 53–63]. Both in their roles as DNA elements or as expressed ncRNAs, TEs play pivotal regulatory roles in a wide range of brain tissues [14, 41, 64–75], primarily via local (cis) regulation of neighboring genes, rather than through the regulation of distant genes (trans) [76–79].

Several studies have considered the potential effects of TEs as either genetic (DNA) and/or genomic (ncRNA) risk factors in the etiopathogenesis of neuropsychiatric disorders, such as schizophrenia [64, 80–85], autism spectrum disorders [86–88], Rett syndrome [74, 89, 90], and others (see [66, 91] for reviews). TE-mediated mechanisms, together with correlated chromatin modifications/chromatin decondensation, have also been invoked as contributing to cellular aging and neurodegenerative disorders [5, 6, 92–99]. Studies in Alzheimer’s disease, and other tauopathies such as progressive supranuclear palsy (PSP), have shown alterations in TE expression profiles that suggest a potential involvement in Tau-dependent pathological mechanisms leading to neurodegeneration [2, 100]. Widespread Tau-dependent chromatin decondensation leads to the re-expression of otherwise silenced TEs, without re-activating TE retrotransposition [98]. Such a Tau-induced expression of TEs, mostly long interspersed nuclear elements (LINEs) and human endogenous retroviruses (HERVs), has been associated with cognitive decline in manifest AD, in association with increased neurofibrillary tangles (NFTs) found in post-mortem AD brains, and in support of a proposed pathogenic role of TEs in neurodegeneration [101].

To further assess the mechanistic role of TEs in aging-related processes, a recent study examined their expression profile within a senescent-associated secretory phenotype (SASP), a cellular stage characteristic of senescent cells secreting high levels of inflammatory markers [92]. TEs are abnormally activated in SASP and appear to be responsible for the chemically induced inflammatory cascade that recruits the immune response, and ultimately results in the elimination of SASP-expressing cells (both neurons and glia) [3, 92, 102]. Finally, RNA transcripts from HERV-K elements can bind to and activate Toll-like receptor 8, leading to neuronal apoptosis via TLR and SIRM1 signaling [103], or express a novel viral protein (cryptically encoded within the HERV-K *env* transcript) that shows neurotoxic properties [104]. Taken together, these investigations reveal a plausible role for TEs in the neuroinflammatory and immune-mediated pathological aging processes, possibly leading to frank neurodegeneration. Interestingly, this expanding evidence relating TEs to neurodegeneration has prompted some to hypothesize using TE expression profiles as markers of aging, and to aid in diagnostic accuracy [93].

Herein, we evaluate the differential expression (DE) of TEs within a unique sample of subjects from a late-onset Alzheimer’s disease (LOAD) cohort for which we have RNA-sequencing data obtained before and after their *pheno*conversion from the presymptomatic to the symptomatic forms of the disease [105], using our validated RNA-based analytical pipeline [64]. Through such a quantitative characterization of the DE TE loci within the human genome, we wished to evaluate whether such blood-derived TEs might provide robust biomarkers for cognitive decline and/or the *pheno*conversion to the manifest stages of LOAD.

## Materials and methods

### Subjects and study design

The overall study population providing specimens for this study has been previously described [105, 106]. In brief, subjects were independent, community-dwelling older adults, aged ≥ 75 years, without known diagnosis of Alzheimer’s disease (AD) or mild cognitive impairment (MCI) or other major neurological or medical illnesses. Each subject underwent a fasting blood draw and thorough neuropsychological testing at the time of entry and yearly thereafter, for a maximum of 6 visits. A total of 525 participants were enrolled over the course of the 5-year study. After year 3 of the study, a biomarker discovery cohort of participants that met strict neuropsychologically defined criteria for either normal cognition (NC), newly diagnosed amnestic MCI (aMCI), or AD were defined. In addition, a group of participants were identified as entering the study with normal cognition (NC), but over the course of the study developed criteria for amnestic MCI (aMCI) and/or AD (*pheno*converters; Converters). These latter individuals were designated as Converter_pre_, while meeting NC criteria, and Converter_post_, once meeting the neuropsychologically defined cognitive criteria for either aMCI or AD. A biomarker validation cohort was similarly defined at the end of the study. Details of the cognitive assessment and operationalization of clinical criteria used to define the groups can be found in our previous publication [105]. The Institutional Review Boards (IRBs) at both the University of Rochester, NY, and the University of California at Irvine (CA) approved a common research protocol for this investigation. The Georgetown University Medical Center (GUMC, Washington, DC) IRB, which had approved the biorepository for collected specimens for this clinical investigation, also approved this common research protocol. Written informed consent forms were discussed with subjects at the time of entry into the study and all subjects entered into this investigation gave verbal and written consent.

### RNA sample collection, processing, and storage

Prior to blood collection, the participant’s height, weight, blood pressure, pulse, temperature, list of current medications, and whether food or drink other than water before midnight had been consumed were recorded. The date and time of the blood collection were also recorded, and in the case of multiple blood tubes being collected, PAXgene™ RNA tubes (# 762,165, BD Diagnostics, Sparks, MD, USA) were drawn last. Blood samples for RNA isolation were collected into three 2.5-ml PAXgene™ RNA tubes, inverted 10 times and stored at room temperature for a minimum of 2 h (Note: once blood is collected and mixed thoroughly within PAXgene™ tubes, samples are stable for 72 h at 18–25 °C). Following the initial period of incubation, PAXgene™ tubes were placed on wet ice and shipped by priority overnight delivery from the clinical collection site (at the U of R or UCI) to the GUMC Neuroscience Biorepository. All received and accepted samples, including all PAXgene™ tubes, were transported from collection to the Biorepository within 24 h. Upon arrival, PAXgene™ tubes were inverted 10 times and stored at − 20 °C, if not immediately processed for RNA. Total RNA was extracted using the PAXgene Blood RNA Kit (# 762,164, Qiagen, Inc., Germantown, MD, USA), according to the manufacturer’s instruction. Frozen PAXgene™ tubes were left to thaw at room temperature for 2 h prior to RNA processing. The isolated blood-derived RNA was quantified using a NanoDrop ND-1000 spectrophotometer (Thermo Fisher Scientific, Inc., Waltham, MA, USA), cataloged, and stored at − 80 °C until ready for further analysis. Total RNA specimens from selected subjects were shipped frozen (on dry ice) to Expression Analysis Inc. (EA, a Quintiles Company, Durham, NC, USA) by priority overnight delivery for RNA-sequencing (RNA-Seq).

### RNA expression analysis methods

EA performed RNA-sequencing (RNA-Seq) upon receipt of the frozen specimens, using their proprietary methods, and using an Illumina High Seq sequencing platform. Briefly, after specimen thawing, globin mRNA was depleted from the total RNA samples using the GLOBINclear-Human Kit (# AM1980, Life Technologies, Grand Island, NY, USA), according to vendor protocol. A total of 1.25 µg of RNA isolated from whole blood was then combined with biotinylated capture oligonucleotides complementary to globin mRNAs. The mixture was incubated at 50 °C for 15 min to allow duplex formation. Streptavidin magnetic beads were added to each specimen, and the resulting mixture was incubated for an additional 30 min at 50 °C to allow binding of the biotin moieties by streptavidin. Complexes comprised of streptavidin magnetic beads bound to biotinylated capture oligonucleotides that are specifically hybridized to the specimen globin mRNAs, and were then separated from the specimen using a magnet. The globin-depleted supernatant was transferred to a new container and further purified using RNA binding beads. The final globin mRNA-depleted RNA samples were quantified using a NanoDrop ND-8000 spectrophotometer (Thermo Fisher Scientific, Inc., Waltham, MA, USA), and assessed for RNA integrity using an Agilent 2100 BioAnalyzer (Agilent Technologies Inc., Santa Clara, CA, USA) or Caliper LabChip GX (PerkinElmer, Waltham, MA, USA). RNA samples with A260/A280 ratios ranging from 1.6 to 2.2, with RIN values ≥ 7.0, and for which at least 500 ng of total RNA was available proceeded to library preparation. Libraries were then prepared for RNA-Seq using the TruSeq RNA Sample Prep Kit (Illumina, Inc., San Diego, CA, USA), including the use of Illumina in-line control spike-in transcripts. Library preparation was initiated with 500 ng of RNA in 50 µl of nuclease-free water, which was subjected to poly(A) + purification using oligo-dT magnetic beads. After washing and elution, the polyadenylated RNA was fragmented to a median size of ~ 150 base pairs (bp), and then used as a template for reverse transcription. The resulting single-stranded cDNA was converted to double-stranded cDNA, with ends repaired to create blunt ends, and then, a single A residue was added to the 3ʹ ends to create A-tailed molecules. Illumina indexed sequencing adapters were then ligated to the A-tailed double-stranded cDNA. A single index adapter was used for each sample. The adapter-ligated cDNA was then subjected to PCR amplification for 15 cycles. This final library product was purified using AMPure beads (Beckman Coulter, Inc., Pasadena, CA, USA), quantified by qPCR (Kapa Biosystems, Inc., Wilmington, MA, USA), and its size distribution assessed using an Agilent 2100 BioAnalyzer or Caliper LabChip GX. Following quantitation, an aliquot of the individual library was normalized to 2 nM concentration and equal volumes of specific libraries were mixed to create multiplexed pools in preparation for Illumina sequencing, performed at 75 cycles of paired end sequencing to reach a minimum of ~ 50 million reads/subject and to detect also low abundance transcripts. We obtained between 68 and 109 M reads/subjects representing more than 40-fold enrichment for target sequences.

We performed Quality Controls for all the RNA-Seq data using fastqc (https://www.bioinformatics.babraham.ac.uk/projects/fastqc/) and also checked for the possible presence of batch effects or other confounding variables using Surrogate Variable Analysis (SVA/svaseq: [107]).

### RNA-Seq data files, retrieval, and storage

EA delivered two portable hard drives to the GUMC Biorepository, containing the following RNA-Seq analysis data files: FASTQ; BAM; translated CEL; quality control; and summary. FASTQ and BAM files were uploaded by file transfer protocol (FTP) using the Amazon Simple Storage Service (S3) offered by Amazon Web Services (AWS) for cloud computing. All EA RNA-Seq data files are currently backed up at the Georgetown University Information Services Laurel data center.

### RNA-Seq data analysis

Initially, extracted FASTQ files from the provided Illumina BAM files, per the provider instructions, generated a pair of forward and reverse reads for each subject specimen. To map and quantify the level of expression for each discrete TE at their unique genetic locations, we applied a TE RNA-Seq pipeline that was previously developed and experimentally validated (Guffanti et al. [64]). Our RNA mapping strategy for TEs is based on modifications of the Trinity Genome Guided (GG) assembly protocol [108, 109].

In a first step, using the sequencing aligner HISAT2 [110], we align raw RNA reads to the TE reference genome, which was extracted from the Repbase/RepeatMasker database (v4.1.0) of the human genome version GRCh38. In this first step, the goal is to sort out the reads that potentially map to the TE reference genome and discard the reads that do not. The selected reads are then separately submitted to the Trinity-GG algorithm that assembles these reads into transcripts that represent the de novo assembled transcriptome for TEs. Using Megablast, each de novo assembled TE transcript was aligned to the RepeatMasker reference, and we filtered out all transcripts that show less than 95% identical matches and that align for less than 90% of their length with the reference TE. The expression of each discrete TE transcript was quantified using Kallisto (v.0.43.0) [111], generating matrices with TPM (transcript per million) values, where TPM is the transcript count of each TE divided by the sum of the transcript counts of each sample, multiplied by one million. TPMs were cross-sample normalized for subsequent analyses using the TMM (trimmed mean of *M* values) normalization approach, using edgeR. For each test sample, TMM normalization is computed as the weighted mean of the log expression ratios between a reference and a test sample, after exclusion (trimming) of the most expressed transcripts and the transcripts with the largest log ratios [112]. To test for differential expression of TEs in pre/post conversion samples, we used the EdgeR Bioconductor package [113, 114] which keeps only those transcripts that have at least 1 read per million in at least 2 samples, and used the differential analysis of sequence read count data for paired samples for the comparison of data before and after the phenotypic onset of AD. Because individual TE transcripts could align with more than one reference TE locus, we implemented a sequence alignment strategy designed to univocally identify discrete TE-encoded transcripts that are stringently aligned to their unique *primary* genomic locations. It was required that transcripts must align with a TE reference sequence for at least 90% of the transcript length, and show at least 95% sequence identity between the sequence of each candidate TE-derived transcript and the matched reference TE sequence from RepeatMasker.

Since Converter_pre_ and Converter_post_ designations correspond to different states/phases of the same individuals, differential expression between them was evaluated using edgeR with a paired-sample approach. First, a design matrix was generated without an interaction term. This was then applied to a generalized linear model to normalize expression data. Finally, likelihood ratio tests were performed for Converter_post_ vs. Converter_pre_ samples.

### In silico* analyses of TE-mapping transcripts*

To investigate the chromatin states of the differentially expressed TEs in our sample, the Core 15-state model from the Epigenomics Roadmap website (https://egg2.wustl.edu/roadmap/web_portal/chr_state_learning.html#core_15state) was used.

Hg38 coordinates were converted to hg19 coordinates using the liftOver Bioconductor package (Bioconductor Package Maintainer (2019). liftOver. R package version 1.10.0. https://www.bioconductor.org/help/workflows/liftOver/), for compatibility with the genome reference of the Epigenomics Roadmap. TEs that mapped in genomic regions that did not successfully convert from hg38 to hg19 were removed. The Bedops software [115] was used to assess the overlap between TE coordinates and chromatin states from 8 different tissues including adult blood, adult brain regions (E062: peripheral blood mononuclear primary cells; E073: brain dorsolateral prefrontal cortex; E072: brain inferior temporal lobe; E067: brain angular gyrus; E071: brain hippocampus middle; E074: brain substantia nigra; E068: brain anterior caudate; E069: brain cingulate gyrus), 3 fetal brain regions (E081: fetal brain male; E082: fetal brain female; E070: brain germinal matrix), and neuronal cultures (E007 and E009: H1 derived neuronal progenitor cultured cells; E010: H9 derived neuron cultured cells). The mix.heatmap function from the CluMix R package [116] was used to generate heatmaps of the Core 15-state model analysis data. Similarities between subjects were measured by Gower’s general similarity coefficient. Similarities between variables were based on distance correlation. Standard hierarchical clustering, with default Ward’s minimum variance method, was applied to obtain dendrograms of the considered subjects. Variations among the considered 14 tissues were represented by applying Kruskal’s non-metric MDS to distance correlation, as implemented in the isoMDS function from the MASS library of R software [117]. Colors for the 15 chromatin states were set using the color codes provided by the Roadmap Epigenomics Project (https://egg2.wustl.edu/roadmap/web_portal/chr_state_learning.html). Tissues with multiple chromatin states were colored in blue and labeled as “Mx” (i.e., mixed chromatin states).

### Constructing time-dependent RNA trajectories

We used the R-Bioconductor package Monocle [118] to analyze time-dependent RNA trajectories and identify the pre to post transition path in the group of individuals that developed LOAD. We performed the analysis of TE RNA transcript expression values to sort individuals in a pseudotime order. After converting TPM values into RNA counts via the *relative2abs* function, implementing the algorithm called Census [119], we normalized the RNA counts across transcripts via the *estimateSizeFactors* and *estimateDispersion* functions, and filtered out transcripts below the expression threshold of 0.1, while retaining transcripts expressed in at least 4 individual RNAs of the data set. The time-dependent trajectory analysis was performed on a set of transcripts selected to be DE at the threshold *q* value < 0.01 between time points comparing transcripts at the Converter_pre_ and Converter_post_ conditions. After applying the data dimensionality reduction, using the Discriminative Dimensionality Reduction with Trees (DDRTree) method, the RNAs of all individuals were ordered along the trajectory using the *orderCells* function. The information on the collection time was leveraged to identify the start point of the pseudotime. Then, the identified start state was used as root to reorder the RNAs. To find transcripts that change as the RNAs make progress along the pseudo-temporal trajectory, we tested for DE transcripts as a function of the pseudotime, recording the progress of each RNA through the developmental path. To identify patterns of covariation of transcripts along the pseudotime, we used the *plot_pseudotime_heatmap* function that generates smooth expression curves for each transcripts and clusters them based on profile similarity.

### Predictive modeling using machine learning (ML)

In addition to the previous analyses, we were interested to identify possible TE biomarkers for the comparisons “Converter_pre_ vs Converter_post_” and “Converter_pre_ vs NC” through a machine learning (ML) technique. Our goal was to identify those TEs that accurately discriminate between the groups of patients and predict their health state with significant accuracy. First, the Shannon entropy value of the expression levels for each TE was calculated, using a function created specifically for this study (see Supplementary Methods). Shannon’s entropy is a measure that estimates the amount of information present within a message. This step allowed us to remove all the TEs from the dataset that did not have a sufficient amount of information. Ten thousand TEs, ranked by the entropy of their level of expression, were retained for further analyses. The data were then organized in a matrix whose rows matched the individual samples and selected TEs were represented in the columns. This matrix was then used for selecting features based on the Boruta algorithm, with the Boruta R package applied to Random Forests [120]. The parameters used to run Boruta were as follows: *p* value =  < 0.05, ntree = 10,000, maxRuns = 100. The remaining functionalities obtained by Boruta were used to discriminate those TEs that were representative for a particular class of patients with respect to any other (e.g., Converter_pre_ vs. Converter_post_ or Converter_pre_ vs NC). Next, our dataset was divided into a training and test set, selecting 70% and 30% of the samples, respectively, using the Caret and Ranger R packages [121, 122] with the “down” parameter set to “TRUE” in order to take into account any variability imbalances within classes. The generalizability of our models was validated using a five times cross-validation and by the “train” function of Caret. Model performance was assessed using standard functions implemented in Caret. The estimation of the AUC values for the Converter_pre_ vs. Converter_post_ and Converter_pre_ vs NC (ROC curves) was generated using pROC R package [123].

### PCA analysis

To validate the results obtained from both RNA-sequencing and ML analyses, a PCA model [124] was applied. Initially, a PCA analysis was run on the gene expression data of the significant DE TEs from the Converter_pre_ vs. Converter_post_ (*n* = 1790) and the Converter_pre_ vs NC (*n* = 503) RNA-sequencing analyses, and then to the gene expression of the TEs (*n* = 24) selected by ML methods for the same comparisons (see Supplementary Methods).

### Function prediction of TEs and gene ontology

To initially evaluate a possible functional role of the 1790 and 503 DE TEs that were significant in the Converter_pre_ vs. Converter_post_ and Converter_pre_ vs NC comparisons, respectively, we looked at the annotations of their neighboring genes within the human genome hg38 with the software GREAT [125]. To constrain our analyses to the hypothesis of a *cis*-regulatory function of TEs, we considered only those protein-coding genes that lie within a distance of 5000 bp, either upstream or downstream of the genomic location for any given TE, using all the transposable elements present in the RNA-sequencing analysis as background. Finally, using multiple annotation sources, an estimate of enrichment was determined for biological and molecular functions for those gene families with identified annotated genes using GREAT (http://great.stanford.edu/public/html/).

## Results

### Identification and quantification of expressed TEs from RNA-Seq data

**Fig. 1:**
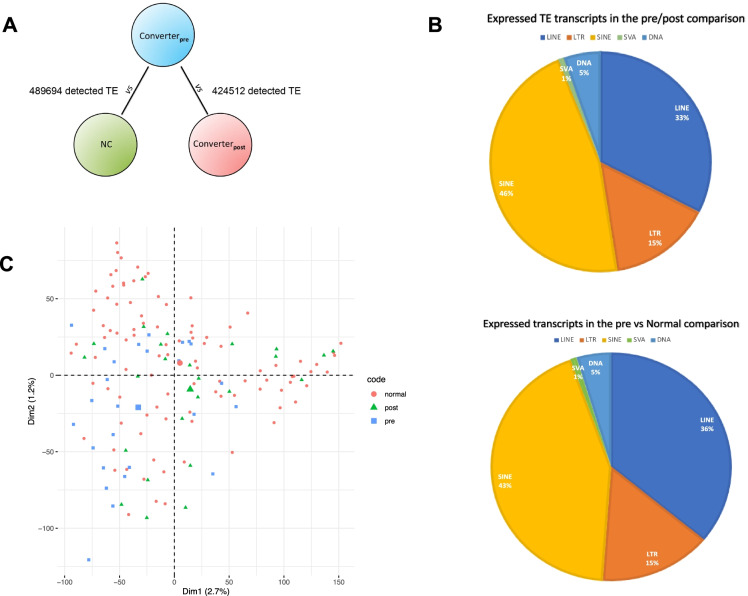
**A** A graphical representation of the comparisons with the QC numbers of observed TE-mapping transcripts in the Converter_pre_ vs. Converter_post_ and Converter_pre_ vs NC samples. **B** The relative proportion of expressed TEs by classes in the 2 comparisons. **C** The first 2 dimensions of PCA for normal, pre, and post subjects that do not present any preferential subclustering (see also text for more details)

To quantify the expression of TEs and detect their differential expression for the comparisons of interest, a transcriptome assembly and annotation pipeline was applied to process raw RNA-Seq data with a *G*enome-*G*uided *d*e novo *a*ssembly (GGdna) workflow [64, 126]. Our pipeline allows detecting expression of each single TE at its genomic location across the whole human genome, thus yielding a granular analysis of the expression of precisely mapped elements. We applied our GGdna pipeline to more than half a billion reads, which had an average sequence length of ~ 150 nucleotides and an average read quality of 39.3. RNA transcriptomes were sequenced from the whole blood samples of 25 individuals before (Converter_pre_) and after (Converter_post_) their *pheno*conversion to manifest amnestic MCI (aMCI) or late-onset Alzheimer’s disease (LOAD), and from an independent subgroup of 64 age- and sex-matched controls that have retained normal cognition along the whole 5 years of observation (normal cognition (NC)). The average age of the 25 individuals who developed aMCI/LOAD is 81.2 (± 4.1) years (14 females and 11 males), averaging 2.1 (± 1.1) years to the *pheno*conversion. The 64 subjects of the NC group (43 females and 21 males) had an average age of 81.6 (± 3.9) years. These 89 subjects are part of a larger sample for which RNA data are available. Based on quality measures (see “Materials and Methods”), 799,853 and 624,793 RNA transcripts were retained from the Converter_pre_ vs. Converter_post_ and Converter_pre_ vs NC comparisons, respectively. A QC analysis using *fastqc* did not show abnormalities due to RNA storage and sequencing. All transcripts are putatively mapping to the reference sequences of discrete TEs reported in RepeatMasker/Repbase (v 4.1.0). After further QC to remove transcripts that are mapping to multiple locations within the genome (see “Materials and Methods”), the number of transcripts reduced to 424,511 (Converter_pre_ vs. Converter_post_) and 489,694 (Converter_pre_ vs NC) elements, aligning to 338,447 and 373,159 unique reference TE loci, respectively (Fig. 1; Supplementary Table 1).

The mean length of the RNA transcripts mapping the TEs is 377.3 nucleotides (nt) (± 200.9) and 358.1 nt (± 200), with a mean log counts per million (logCPM) ranging from 5.2 to 7.9. The proportional distribution of expressed transcripts according to TE classes is reported in Fig. 1B. Approximately 10% of the 338,437 and 14% of the 373,159 uniquely mapped reference TE loci belong to evolutionary recent TE families [127–134]. Assuming a *cis*-regulatory effect of TEs, there are 22,445 and 23,020 unique genes that are putatively controlled by these expressed TEs, of which 14,544 and 14,765 are reported as protein-coding genes by the current hg38 annotation in the pre/post and pre/normal comparisons respectively. A principal component analysis (PCA) did not reveal a clear separation of Converter_pre_, Converter_post_, and NC samples, indicating that no systematic differences in TE expression exist among the 3 groups (Fig. 1C). A further analysis looking at possible confounders (SVA: Surrogate Variant Analysis) did not detect any significant effect. For both the Converter_pre_ vs. Converter_post_ and Converter_pre_ vs NC comparisons, we thus proceeded to identify differentially expressed (DE) TEs and evaluate their distributions across TE classes and families.

### Differential analysis of TE expression in pre vs post and pre vs normal

**Fig. 2:**
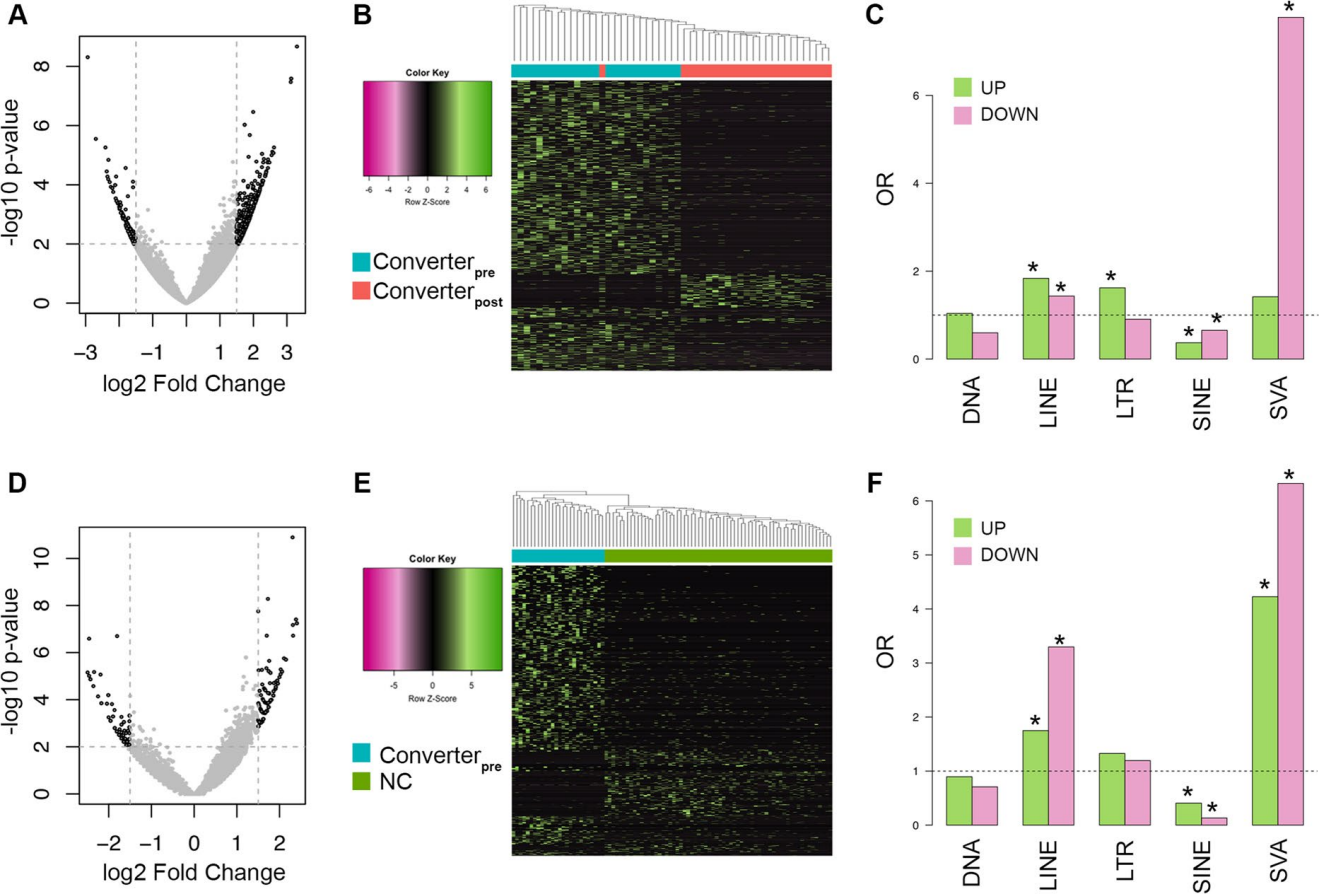
Differential analysis of TE expression. **A** Volcano plot of the results from the differential expression analysis of TE transcripts in the Converter_pre_ vs Converter_post_ comparison. Significant RNA transcripts at logFC ± 1.5 and *p* value ≤ .01 are highlighted in black. **B** Scaled heatmap and unsupervised hierarchical clustering of the log2 TMM values of the 1790 TEs identified as differentially expressed in the Converter_pre_ vs Converter_post_ comparison. Samples are annotated with different colors according to the group (Converter_pre_ or Converter_post_). **C** Enrichment analysis for up- and down-regulated differentially expressed TE transcripts according to their class. Stars mark significantly enriched TE classes (Fisher’s exact test *p* value ≤ .01). **D**, **E**, **F** The panels reports the same plots described above, but for the Converter_pre_ vs NC comparison

Using conservative significance criteria (nominal significance *p* value < 0.01, logFC >  + 1.5), we found 1790 transcripts mapping to reference TEs that were DE between Converter_pre_ and Converter_post_ samples: 1543 (86%) with higher expression values and 247 with a lower expression values in Converter_pre_ than in Converter_post_ states (Fig. 2 A and B; Supplementary Table 2). Up-regulated DE TEs are significantly enriched in LINE and long terminal repeat (LTR) elements, while down-regulated DE TEs are enriched in LINE and composite repetitive element (SVA) named after its three main components, short interspersed nuclear elements (SINE), variable number of tandem repeats (VNTR), and Alu elements (Fig. 2C). Both up- and down-regulated DE TEs were depleted in SINE (Alu) elements (Fig. 2C). Within the over-expressed TEs, 70 are evolutionarily recent LINE1 (L1HS, L1P1/2/3 or L1PA2-4) and at least one L1HS element on chr6:24,811,658–24,817,706 is insertionally polymorphic, with highest frequencies in African populations (1000 genomes: YRI, 85.42%; LWK, 84%; CEU, 46%; CHB, 56%; ITU, 56%, A. Boattini, personal communication), and putatively acting as a weak enhancer of the RIPO2 gene. We also observed 24 evolutionary recent HERVs (HERVK-int, LTR5_Hs, LTR7) and 18 SVAs. Within the under-expressed TEs, LINE elements are a mix of evolutionarily recent and old elements, and 10% of LTRs are represented by HERVK family elements (Supplementary Table 2). Importantly, we cannot exclude a priori that these 1790 TEs have been identified as differentially expressed in the Converter_pre_ vs Converter_post_ comparison because of the longitudinal design of the study (i.e., these TEs have an age-dependent expression), independently of the conversion to aMCI/LOAD. To rule out this possibility, we evaluated whether the 1790 DE TEs showed an age-dependent expression in the NC group. NC subjects did not cluster according to their age for these TEs, and accordingly the first two components of the PCA calculated on the expression values of the 1790 TEs did not show association with age in the NC group (Supplementary Fig. 1). Collectively, these observations suggest that the differential expression of the 1790 TEs that we identified in the Converter_pre_ vs Converter_post_ comparison cannot be simply ascribed to the fact that subjects were evaluated at two timepoints.

The analysis of RNA transcripts contrasting Converter_pre_ and NC subjects yielded 503 DE TEs, of which 383 and 120 were over- and under-expressed in the Converter_pre_ compared to NC group, respectively (Fig. 2 D and E; Supplementary Table 3). The pattern of enrichment across TE classes was similar to that observed in the Converter_pre_ vs Converter_post_ comparison: over-expressed DE TEs preferentially mapped LINE and LTR but not SINE elements, while under-expressed DE TEs were enriched in LINEs and SVAs and again depleted in SINEs (Fig. 2F; Supplementary Table 3).

### *Relationship between Converter*_*pre*_* vs Converter*_*post*_* and Converter*_*pre*_* vs NC comparisons*

**Fig. 3:**
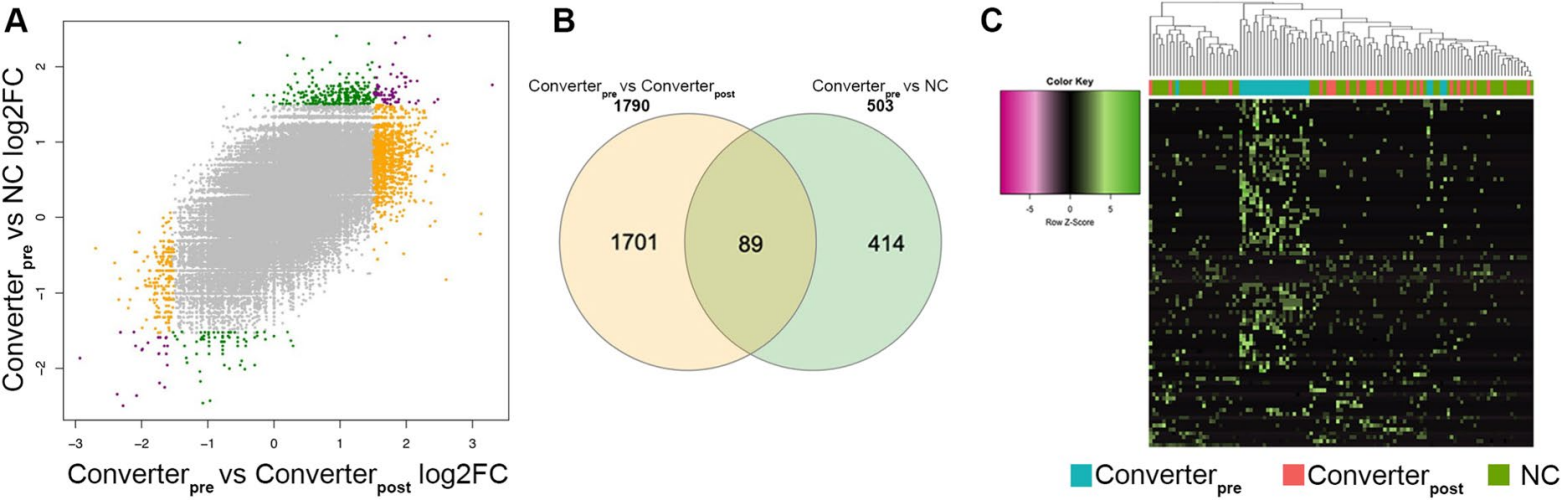
Relationship between Converter_pre_ vs Converter_post_ and Converter_pre_ vs NC comparisons. **A** Correlation between the log2 fold changes (log2FC) of the expressed TE from the Converter_pre_ vs Converter_post_ and Converter_pre_ vs NC comparisons. TEs significant in the Converter_pre_ vs Converter_post_ comparison are highlighted in yellow, and TEs significant in the Converter_pre_ vs NC comparison are highlighted in green, while the 89 TEs significant in both the comparisons are highlighted in purple. **B** Venn diagram showing the intersection between DE TEs significant in Converter_pre_ vs Converter_post_ and Converter_pre_ vs NC comparisons. **C** Scaled heatmap and unsupervised hierarchical clustering of the log2 TMM values of the 89 TEs common to Converter_pre_ vs Converter_post_ and Converter_pre_ vs NC comparisons. Samples are annotated with different colors according to the group (NC, Converter_pre_, or Converter_post_)

As described in the previous paragraph, we found a higher number of DE TEs in the Converter_pre_ vs Converter_post_ compared to the other comparison. To gain better insights into the relationships between the 3 groups, we investigated whether changes in TE expression were shared between the different comparisons. There was a clear positive correlation between the fold changes (FC) resulting from the Converter_pre_ vs NC and those obtained from the Converter_pre_ vs Converter_post_ comparisons (Fig. 3A). Moreover, we found that 89 TEs were identified as DE in both comparisons (Fig. 3B) and that the extent of this intersection was greater than expected by chance (Fisher’s exact test *p* value < 0.01). All the shared DE TEs showed the same direction of change in Converter_pre_ condition: 66 were over-expressed in both Converter_pre_ vs. Converter_post_ and Converter_pre_ vs. NC comparisons (that is, are upregulated at the Converter_pre_ stage and have lower expression values at both NC and Converter_post_ conditions), while 23 were under-expressed in both comparisons (that is, are down-regulated at the Converter_pre_ stage and have higher expression values at both NC and Converter_post_ conditions) (Supplementary Fig. 2). Unsupervised hierarchical clustering using the 89 TEs (Fig. 3C) shows that Converter_pre_ subjects are uniquely clustered together, while Converter_post_ and NC subjects are more dispersed. Collectively, these results suggest that the Converter_pre_ state is characterized by a specific TE expression profile (or signature) that enables it to be distinguished from both NC and Converter_post_ individuals.

These initial findings suggest a biological, preclinical difference exists between subjects that are phenotypically normal (i.e., NC vs. Converter_pre_), making possible to distinguish between those that will likely remain healthy for up to 5 years (the NC subjects) and those destined to develop LOAD within a 12- to 48-month interval (aka, the 25 Converter_pre_ subjects that *pheno*converted to manifest LOAD). The large number of DE TEs that we observe in these Converter_pre_ subjects during their transition to manifest LOAD suggests that their genomes are experiencing extensive dysregulation of TEs that are putatively controlling expression of specific protein-coding genes before the onset of the disease. A PCA of the Converter_pre_ vs. Converter_post_ samples and their significant DE TEs shows a wide dispersion of data on the first 2 PCA dimensions for the Converter_pre_ condition and a stunningly compact degree of clustering for their Converter_post_ condition (Supplementary Fig. 3A). In contrast, all the NC subjects are evenly distributed along the intersection of the PCA dimensions (Supplementary Fig. 3B).

Comparing NC vs Converter_post_, we found 344 DE TEs, 62 of which are in common with the Converter_pre_ vs Converter_post_ comparison and 16 with the NC vs Converter_pre_ comparison (Supplementary Fig. 4). We did not find any TE common to the 3 comparisons. This observation suggests that there is not a clear progression in TE expression changes from NC to Converter_pre_ to Converter_post_ conditions. On the contrary, some TEs are specifically deregulated only in Converter_pre_ condition, and their altered expression is not maintained (or at least, it is not statistically significant in our dataset) in the Converter_post_ condition, i.e., in the overt disease. In parallel, some TEs are already deregulated in the Converter_pre_ condition and maintain a similar altered expression in the Converter_post_ condition.

### Time-dependent analysis with Monocle

**Fig. 4:**
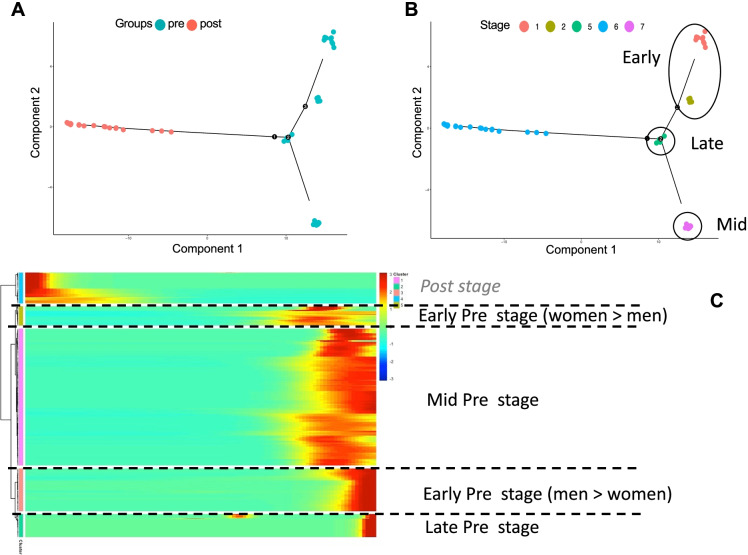
**A** and **B** show the pseudotime continuum from a Converter_pre_ (dots on the right side) to a Converter_post_ (dots on the left side) for the subjects that developed AD during the period of observation. Dots represent subjects: in **A**, blue dots are subjects at their Converter_pre_ condition and red dots are those at their Converter_post_ condition. In **B**, blue dots show the Converter_post_ condition for subjects; the other colors show different Converter_pre_ stages. **C** A heatmap expression matrix for significant DE TEs at the 3 (early, mid, and late) Converter_pre_ stages in addition to the Converter_post_ phase

To better exploit the longitudinal design of our study, we analyzed how the TE transcriptional activity developed across time during the transition from Converter_pre_ to Converter_post_ using Monocle 2 [118, 119]. TE transcriptional profiles appeared to cluster according to the individual RNAs’ collection time and scattered along the temporal trajectory of *pheno*conversion to LOAD, in a pseudotime-dependent manner (Fig. 4A). Overall, the transition from being in a Converter_pre_ into a Converter_post_ state requires about 45 “pseudotime” discrete units, representing a striking approximation of the observed 12 to 48 months that these subjects required for their clinical *pheno*conversion. Such TE transcriptional changes lead to clustering of Converter_pre_ RNAs at the very beginning of the pseudo-temporal trajectory while the Converter_post_ RNAs are distributed at the opposite extreme of the pseudo-temporal path to *pheno*conversion (Component 1 in Fig. 4 A and B). This temporal distribution reflects the patterns of transcriptional activation of classes of TEs at the Converter_pre_ and Converter_post_ stages of LOAD development. The pseudo-temporal reconstruction shows that TEs change consistently with the development of LOAD across individuals, mimicking almost entirely the expected timing of the transition from Converter_pre_ to Converter_post_ stages within these particular individuals. Notably, there is a remarkable differentiation of the pseudo-temporal starting points across the Converter_pre_ stage(s), with individuals clustering at different positions along Component 2 of Fig. 4 A and B, suggesting a degree of heterogeneity of TE-identified Converter_pre_ conditions across individuals (Fig. 4B).

Once we ordered the Converter_pre_/Converter_post_ individuals in stages of disease development, we sought to identify which TEs dynamically change as a function of disease stage when the individual RNAs progress through disease development. For each TE, we modeled its expression by fitting two models (full and reduced) that differ based on whether the individual RNA classifications are explicit or not [119] (Fig. 4C). A total of 2408 TEs appeared regulated during the transition to a clinically evident (manifest) phase of LOAD. We further explored whether specific families of TEs are more highly expressed in an individual’s Converter_pre_ RNA type compared to another, and whether specific classes of TEs tend to be co-expressed along the pseudo-temporal trajectory. At the threshold of false discovery rate (FDR) < 0.01, the Monocle cluster analysis identifies 5 groups of TEs that display patterns of similar expression within each cluster, but no TE classes or families appeared enriched across these 5 clusters. The partition of Converter_pre_ individual RNAs into separate subgroups seems rather consistent with differing preliminary stages of progression of TE activation to manifest stages of LOAD (Converter_post_). The analysis of the pseudo-temporal trajectory indicates that the TE transcriptional activity delineates three different branches within global Converter_pre_ TE transcripts. This finding appears dependent on the particular time an individual is analyzed, prior to the onset of disease at his/her own Converter_pre_ stage (time), and shows consistency in partitioning the Converter_pre_ stage into early, mid, and late phases, with each phase signaling the time to AD onset (early: 36–48 months; mid: 18–36 months; late: < 18 months) (Fig. 4B). A total of 1006 TEs characterize these 3 phases of the Converter_pre_ state along a “dynamic” pseudotime trajectory, with 106 TEs overlapping with those found significant as DE TEs in the Converter_pre_ to Converter_post_ comparison (Fig. 4C; Supplementary Table 4). The early phase is also characterized by a sex effect, further generating two subgroups with different women:men ratios. Such finding suggests the presence of heterogeneity at the “pre” stage, due to both sex and time-to-disease-onset effects. Each individual at his/her Converter_pre_ state is thus characterized by a specific TE signature that marks his/her progression toward the Converter_post_ state, which does not show signs of heterogeneity related to TEs’ expression (Fig. 4B).

### Epigenomic landscape of DE TEs in blood and brain tissues

To further characterize these potential peripheral blood biomarkers of early neurodegeneration, we also considered the chromatin states of the genomic regions harboring the DE TEs, both in blood and brain tissues, according to Epigenome Roadmap data. We used the Core 15-state model, in which 5 chromatin marks (H3K4me3, H3K4me1, H3K36me3, H3K27me3, H3K9me3) are combined to predict 15 possible chromatin states, indicative of the biological function of the underlying genomic region [135].

**Fig. 5:**
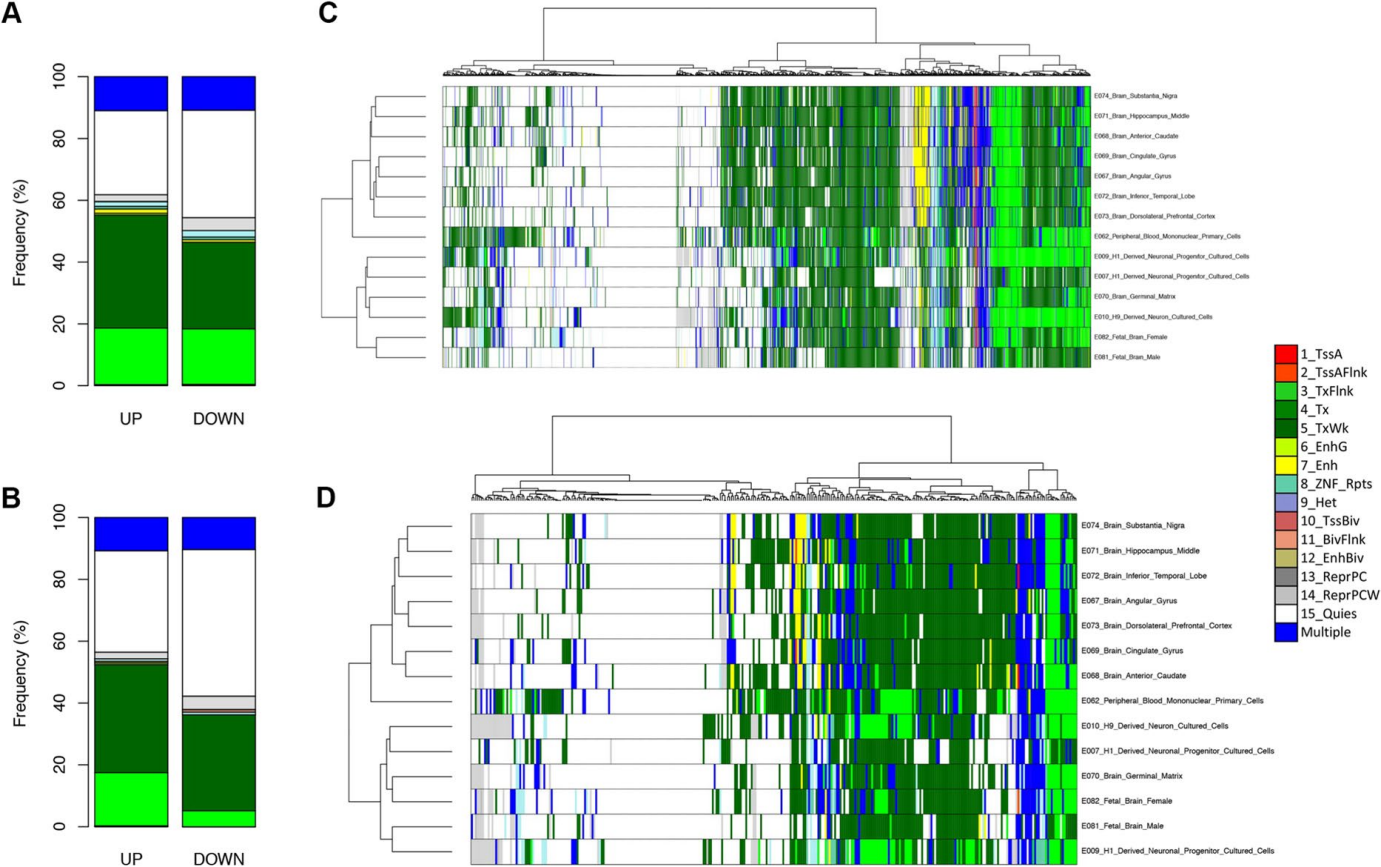
Chromatin states of DE TE. **A**, **B** Distribution of up- and down-regulated DE TE across the chromatin states included in the Core 15-state model in blood cells, considering Converter_pre_ vs Converter_post_ (**A**) and Converter_pre_ vs NC (**B**) comparisons. **C**, **D** Heatmaps with unsupervised clustering of the chromatin states in blood cells, and adult and fetal brain tissue considering the genomic regions overlapping with DE TEs from Converter_pre_ vs Converter_post_ (**C**) and Converter_pre_ vs NC (**D**) comparisons. In all the plots, colors of the chromatin states are shown in the legend and correspond to those used in the Epigenomic Roadmap website; DE TEs whose genomic location encompasses multiple chromatin states are colored in blue

For the Converter_pre_ vs Converter_post_ comparison, we found that 67% of the over-expressed transcripts and 56% of the under-expressed transcripts overlap or intersect with signatures of functionally active chromatin states in blood cells (Fig. 5A). Interestingly, we found that over-expressed TEs in the Converter_pre_ vs Converter_post_ comparison are significantly enriched in TxWk (actively transcribed) and Enh (enhancer) chromatin states (Supplementary File 1). For the Converter_pre_ vs NC comparison, 62% of the over-expressed DE TEs mapped in genomic regions with active chromatin marks in blood cells, while down-regulated TEs mapped mainly to inactive regions and only 40% of down-regulated DE TEs mapped in active chromatin regions (Fig. 5B).

We then considered the chromatin state of the DE TEs using the Epigenome Roadmap Core 15-state model from brain regions. We found that adult brain tissues and fetal brain/germinal tissues, despite organizing in two distinct clusters, show a rather similar profile of active and quiescent chromatin regions [136] to those characterizing the peripheral blood cells (Fig. 5C, D). This observation suggests that at least a fraction of the DE TEs that we identified in whole blood have a similar epigenetic regulation in brain tissues. Indeed, we found that 61% of the DE TEs in the Converter_Pre_ vs Converter_Post_ comparison and 67% of the DE TEs in the Converter_Pre_ vs NC comparison were also expressed in the human dorsolateral prefrontal cortex (DLPFC) using previous data generated by our lab [64] (Supplementary Tables 2 and 3).

Most of the chromatin regions that overlap with the significant DE TEs and presenting with an active Core 15-state model (suggesting a possible functional role as either enhancers or promoters) are also functionally active within adult brain tissues as well as fetal brain tissues. In the Converter_pre_ vs. Converter_post_ comparison, 11 DE TEs are marked as enhancers (m7_Ehn, yellow) in all adult brain tissues, but not in peripheral blood. Some of these DE TE insertions map onto genetic regions linked to Alzheimer’s disease (Supplementary Table 5). For example, a LINE2 on chr1:10,075,287–10,075,497 maps within the second intron of the UBE4B gene [137], and a LINE2 on chr7:105,246,376–105,246,653, found in the NC vs Converter_pre_ comparison, lies within the SRPK2 gene [138]. Moreover, a LINE1 on chr6:36,594,353–36,605,600 in Converter_pre_ vs Converter_post_ comparison was found to be transcriptionally active (m1_TssA, red) in all adult brain tissues, and maps within the SRSF3 gene, known to regulate the innate immune response in resident microglia [139].

### Gene ontology analysis of DE TEs

Expressed TEs can provide different functional roles, whose detailed analyses are beyond the scope of this present work. Herein, however, we performed an exploratory gene ontology analysis, assuming that expressed TEs may work as “*cis*” rather than “*trans*” elements, and thereby regulate the expression of local protein-coding genes. Supplementary Tables 6 and 7 provide the lists of TE *cis* mapped genes that we have used as input lists for either the Converter_pre_ vs Converter_post_ and the NC vs Converter_pre_ pathway analyses (see “Materials and Methods”).

Interestingly, the gene ontology analysis performed using GREAT, on the genes located within 5 kb both up- and downstream of the highest dysregulated TEs in the Converter_pre_ vs Converter_post_ comparison, shows that the most enriched biological families (adjusted *p* value < 0.01) were related to molecular pathways already known to be involved in AD, such as “negative regulation of autophagosome,” “negative regulation of autophagy,” and “positive regulation of dopamine receptor signaling.” Instead, the most enriched gene families in the NC vs. Converter_pre_ comparison show an involvement in the “cellular protein modification process,” “protein modification process,” and “macromolecule modification” (with enrichment in molecular functions related to “regulation of skeletal muscle fiber development,” “regulation of myotube cell development,” and “negative regulation of proteasomal activity”).

### Machine learning results 

Using a machine learning (ML) approach, we obtained eight predictive biomarkers by comparing the Converter_pre_ vs Converter_post_ states in samples of subjects that phenoconverted to manifest LOAD, producing a classification accuracy of 78% (Table 1).

**Table 1:** TEs selected by the machine learning analysis. The 8 TEs are able to discriminate Converter_pre_ vs Converter_post_ condition patients with an AUC accuracy of 78%. *Chr*, chromosome; *Start*, TE start position on Chr; *End*, TE end position on Chr; *TE class*, TE class type; *Gene*, gene in which a TE is located

Chr	Start	End	TE	Gene
1	108,926,372	108,927,695	L1M3	GPSM2
1	174,901,607	174,902,942	L1PA10	RABGAP1L/KIAA0471
7	149,486,060	149,486,843	L1ME3D	ZNF746
9	124,885,506	124,887,215	L2a	GOLGA1
11	88,331,047	88,331,958	MER21A	CTSC
17	45,631,040	45,631,664	MER77B	LINC02210
21	15,738,037	15,738,674	L1M5	USP25
X	17,096,688	17,097,359	L1MEd	REPS2

In particular, a L1M5 element is located within the intron of the USP25 gene on Chromosome 21, whose trisomy is associated to Down syndrome (DS; trisomy-21), a condition associated to a high AD risk. USP25 is implicated in activating microglia, and its overexpression allows the de-ubiquitination of a series of molecular substrates that have been associated to synaptic abnormalities and associated cognitive deficits. Removal of USP25 reduces neuroinflammation and rescues synaptic and cognitive functions in a knockout mouse model [140–144]. When analyzing the comparison of Converter_pre_ vs NC individuals, we also found a few significant TEs, most of which are not localized in protein-coding genes and do not have an already known specific relationship with AD. Furthermore, these TEs as biomarkers have an accuracy that is lower (69%) compared with that of the Converter_pre_ vs Converter_post_ condition (Table 2).

**Table 2:** TEs outputted by machine learning analysis. These eight TEs were able to discriminate pre and normal condition patients with an AUC accuracy of 69%

Chr	Start	End	TE	Gene
22	23,900,208	23,900,715	MER9a2	NA
16	67,141,398	67,142,927	MER52A	C16orf70
2	26,305,911	26,306,395	LTR15	AC10896.1
19	11,853,714	11,854,477	HERVK3-int	ZNF439
17	67,398,160	67,399,008	HSMAR1	PITPNC1
2	97,505,313	97,505,837	MER1A	ANKRD36B
20	44,217,986	44,218,725	L1ME4b	OSER1-DT
19	54,668,341	54,669,123	L1M5	LILRB4

**Fig. 6:**
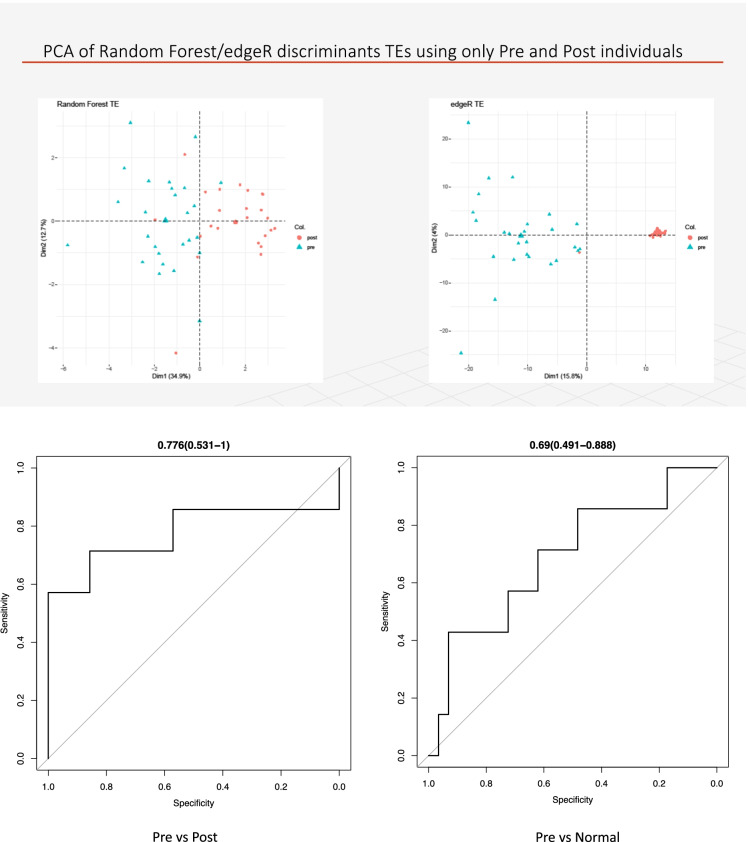
The upper left and right panels show a PCA representation of the accuracy of classification for Converter_pre_ and Converter_post_ subjects, using the 8 selected TEs from Table 1 (left) or the overall 1790 significant TEs (right). The lower panel shows the ROC curves obtained using only the 8 selected TEs from the ML algorithm for Converter_pre_ and Converter_post_ subjects with a correct classification of 78% (left) and the 8 selected TEs for Converter_pre_ and NC subjects with a correct classification of 69%

Finally, PCA analyses and related ROC curves confirmed that both RNA-sequencing DE analyses and the ML approach identified TEs that correctly discriminate between the various patient groups, even when a small number of predictive biomarkers, those selected with the machine learning (ML) algorithm, are used in the models as reported in Fig. 6.

## Discussion

A few published reports have suggested that TEs show a differential expression in patients with Alzheimer’s disease (AD) compared to healthy aged controls, using case–control, retrospective approaches. Here, we have shown that the expression of TEs is massively dysregulated *before* the clinical manifestations of LOAD using RNA-sequencing data at both the preclinical (Converter_pre)_ and clinically manifest (Converter_post_) stages of disease using data from the same subjects. To our knowledge, our analysis is the first of its kind, using data collected from a prospective longitudinal cohort of subjects known to have started in NC state and *pheno*converted to LOAD over a 12–48-month time-frame. *Our prospective design supports the hypothesis that the functional expression of our genome is altered through DE TEs in subjects that are in a preclinical stage of LOAD, when they are otherwise clinically and cognitively undistinguishable by other NC subjects that will not go on to develop the disease*. Our findings suggest that DE TEs may be used as peripheral biomarkers heralding the future development of LOAD within a specific time-frame, although the exact span of such time-frame needs to be more carefully investigated. Our current and experimentally tested time-frame ranges between 12 and 48 months before the clinical onset of the disease, but, at least in principle, subjects that will develop AD at some point in time during their life could present with a TE’s genomic dysregulation even 10 or 20 years (or more) before the clinical onset of AD.

Moreover, many of the DE TEs that we detected in blood leukocytes appear to be functionally expressed enhancers or alternative promoters also in specific brain regions related to AD, using an in silico computational analysis of the Epigenome Roadmap database. These DE TEs that appear also putatively expressed in brain regions are implicated in either memory and/or other cognitive functions (notably, within the hippocampus, the anterior caudate, and the inferior temporal lobe, among others—but see Fig. 5 for a more extended list of brain regions). Thus, our findings might also direct future analyses investigating novel genomic elements that may regulate regional brain genomic mechanisms involved in developing AD. Few previous studies have investigated the possibility to use blood as a surrogate of brain in transcriptomic investigations [145], while more papers evaluated the blood–brain correlation for methylation analyses [146], but to the best of our knowledge, at present there are no studies systematically comparing TE regulation and expression between human brain and blood. It is worth noting, however, that 61% of the DE TE in the Converter_Pre_ vs Converter_Post_ comparison and 67% of the DE TE in the Converter_Pre_ vs NonConverter comparison were also expressed in the human dorsolateral prefrontal cortex (DLPFC) according to previous data generated by our lab (see Supplemental Tables 2 and 5) [64]

Expressed TEs are a large group of genomic elements, collectively classified as ncRNAs. While progressively better identified and known by their genomic locations [7], our current knowledge regarding their functional role(s) remains incomplete. TEs have been considered enhancers or alternative promoters often associated with time- and tissue-dependent regulation of gene expression, as regulators of splicing sites, or contributing to domain rearrangement with preexisting functional elements, producing novel composite architectures via exon shuffling, thereby leading to the genesis of genes with novel functionalities [52]. Additionally, especially in pathological conditions, commonly silenced TEs can be re-expressed due to loss or malfunction of TE-silencing mechanisms. When inappropriately (re-)expressed, TEs can lead to cellular death via multiple mechanisms, but usually involving the direct or indirect activation of the immune system. At present, we do not know whether the DE TEs that we have observed in the development of AD are the *primary* mechanism driving neurodegeneration (etiological agents) or are acting as a *secondary* mechanism (pathogenic elements) unleashed by loss of TE-silencing mechanisms. We have identified, quantified, and evaluated a large number of DE TEs that are nonetheless altering the functional architecture of the genome, under the assumption that expressed TEs act as non-coding RNAs regulating gene expression. Remarkably, other than their better-known role in cancer evolution, TEs have been proposed as pathogenetic elements in various neurological and psychiatric disorders [66, 91], despite our still limited understanding of their specific pathobiologic mechanisms within the brain.

We detected a significant overexpression of LINE1 elements (L1s) prior to the onset of clinical manifestations of aMCI or LOAD, a sort of *LINE1 storm*, adding further support to the potential role of TEs in the genesis of certain neurodegenerative disorders. LINE1 re-expression has already been documented in senescent cells [99, 147] and in the inflammatory and oxidative stress associated with cellular aging, dubbed senescent-associated secretory phenotype (SASP). SASP features an active expression of LINE1 elements and promotes neurodegeneration through the clearance of aging neural and glial cells by the immune system, activated by an unspecified chemical neuroinflammation [92, 93]. As our group and others have noted in SASP or cellular aging, most over-expressed LINE1s are evolutionary recent, with many elements appearing to be human-specific (not shared with other high primates), a finding that has yet to be confirmed by others [93]. Most of our over-expressed LINE1 transcripts overlap with signatures of transcription regulation, as reported in the Epigenome Roadmap data: genic enhancers or Transcription Start Sites (TSS). These signatures of transcription are present in normal blood cells and in both adult and embryonic brain tissues of varying developmental stages.

Noteworthy, 85% of the genes containing LINE1 elements in their ORFs are brain-expressed, according to the Brain Atlas database [148]. We can question whether these over-expressed TE elements could also potentially dysregulate brain genes: 20 of these genes are actually already known to be associated with a “dementia” phenotype and 7 specifically with AD. Whether the TEs that we identified as DE in peripheral blood are also DE and have an effect in the human brain remains an open question. However, under the only functional assumption that we have considered here, that LINE1s can act as *cis* regulators of gene expression, we acknowledge that the genes putatively regulated by these DE LINE1s are also associated with “circadian gene expression” or “interferon-mediated immune response to pathogen-associated DNAs” pathways. Not surprisingly, therefore, one of these genes produces the amyloid precursor protein (APP), and is potentially regulated by a full-length (6025 nucleotides) human-specific L1PA2 element, presenting as an enhancer with a weak-transcription signature. It is tempting to speculate that this finding, if confirmed with larger samples, would support the possibility that APP is at least partially regulated by an L1 element that is undergoing somatic transposition and copy number expansion in AD brains, as a result of LINE1-expressed reverse transcriptase [149–151]—but this finding is still hotly questioned [152]—or via LINE1-mediated overexpression of APP from a germline program [28, 153–160].

Another LINE1 selected by the machine learning predictive algorithm among the 8 TEs that classify Converter_pre_ individuals with 78% accuracy is a L1M5. This L1M5 presents with a signature of a weak enhancer and is located within the first intron of the USP25 gene (formerly known as USP21). The same gene is also tagged by a second LINE1 (a L1M1) in the 4th intron, again showing a signature of a weak enhancer. The USP25 gene has already been shown to be greatly expressed in the brains of DS patients than in controls [161] and overexpression of USP25 in a murine model of DS-AD, particularly in hippocampal CA1 cells, results in microglial activation inducing both synaptic and cognitive deficits [141].

With our current results, we cannot rule out an alternative hypothesis that overexpression of LINE1s could elicit a generalized and unspecific response, like the SASP-induced neuron and glial cell damage previously noted. SASP, or other pathological cell-aging mechanisms, would then activate an immune response that eliminates pathologically aging cells overexpressing LINE1s (including neurons and/or glia). In other neurodegenerative disorders, mechanisms have been proposed by which LINE1s escape silencing and get re-expressed. In both amyotrophic lateral sclerosis (ALS) and frontotemporal dementia (FTD), a clear dysfunction and dislocation of the protein TDP-43 have been identified. Pathologically in over half of these affected patients, TDP-43 is markedly reduced in the neuronal and glial cell nuclei, but instead, accumulated as aggregates within the cytoplasm of these cells in ubiquitinated and hyperphosphorylated forms [162, 163]. This dysfunction, coupled with a 6-nucleotide repeat expansion of the gene C9orf72, induces a massive transposition of LINE1s in both neurons and glia, mediated by disruption of TDP-43 retrotransposon silencing (and by decondensing heterochromatin), which also promotes retrotransposition of other TEs, including LTRs and SINEs, along with LINEs [4, 164, 165].

In addition to LINE1s, we found other DE TEs in our sample set. HERVs are known to be highly expressed in human embryonic stem cells (hESCs), with HERV-H and -K considered markers for pluripotency [166–169]. Progressively silenced during cell differentiation, HERVs still represent one of the largest sources of regulatory elements (mostly enhancers) under both physiological and pathological conditions, and show context-dependent (tissue) specificity [29, 82, 83, 170, 171]. In addition to other neuropsychiatric diseases, HERVs have also been proposed to play a role in neurodegeneration, possibly altering the functional architecture of the genome and contributing to cell death. HERV-K elements can activate Toll-like receptor 8 (TLR8), and lead to neuronal apoptosis via TLR and selective insulin receptor modulator 1 (SIRM1) signaling [103], a shared apoptotic mechanism associated with environmental viral infections (e.g., herpes simplex virus, Epstein-Barr virus). HERV-K may also express a novel viral protein cryptically encoded within their *env* transcript that shows neurotoxic properties [104]. Others have proposed that ERV activation is associated with hippocampus-based cognitive impairment in mice via increased gene and protein expression of the *gag* sequence [172]. Thus, it remains probable, although still speculative, that the role of HERVs in neurodegenerative disorders, and AD in particular, might encompass different mechanisms of action.

In our present analyses, we did not explore any of these specific hypotheses, concentrating instead on generating an extensive catalog of DE HERV and LTR elements prior to the clinical onset of LOAD. Within the 1790 DE TEs identified in the Converter_pre_ vs. Converter_post_ comparison, HERVs/LTRs represent about 25% of the TEs, with not less than 10% being human-specific, and mostly represented by HERV-K elements. Whether HERV-K elements contribute to characterizing certain pathways noted to be enriched in the Converter_pre_ vs Converter_post_ comparison, in addition to the genes putatively controlled by them as regulators, remains uncertain, due to the current imprecise knowledge base for biological effects associated with HERV sequences.

About 50% of the DE SVAs that we detected in the Converter_pre_ vs. Converter_post_ comparison and 35% of those in the Converter_pre_ vs NC comparisons belong to the E and F sub-clades, indicative of the more evolutionary recent SVA elements in our genome [7]. Moreover, they continue to appear to be transpositionally active, or at least can co-mobilize 3ʹ or 5ʹ DNA flanking regions to new genomic loci using TE-mediated transduction [7]. They represent, therefore, one of the most active mechanisms to generate structural variation, if not to generate new gene isoforms (or even new “genes”). Alus, which are significantly depleted in our Converter_pre_ vs Converter_post_ comparisons, seem to act by the same mechanisms observed in SVAs. Thus, these data support the idea that TEs expression, including those associated with SVAs and Alus, are important for risk profiling in preclinical LOAD.

We have shown that TEs can be profiled in a pseudotime model of LOAD development, further suggesting their involvement in a disease fate decision along a pathological continuum. To obtain further insights as to which family of TEs is more highly expressed in the Converter_pre_ vs. Converter_post_ groups, first we examined whether specific classes of TEs are typically co-expressed along the development of the disease. Using a cluster analysis, we found TE expression profiles along pseudotime trajectory cluster according to different stages of the LOAD developmental process. At the threshold of FDR < 1e − 03, the cluster analysis identifies 5 groups of TEs that display patterns of similar expression within each cluster. The unique expression within the Converter_post_ group is clearly different from the 4 associated substages within Converter_pre_. It remains somewhat puzzling as to the significance of the 4 different clusters of subjects defined within the Converter_pre_ stage of disease, although they likely represent clinical heterogeneity. While failing to meet significance due to the limited sample size of our dataset, we also noted that these Converter_pre_ clusters are characterized by a different time-to-disease and a different sex ratio. Such discrimination allows us to define these clusters into two early, a mid, and a late Converter_pre_ transition stage to clinical LOAD. The two early Converter_pre_ clusters are best defined via the women:men ratio, and define subjects at farthest timepoints away from *pheno*conversion to LOAD. Importantly, the four Converter_pre_ clusters display significantly dysregulated TEs (retrotransposon storm) compared to clusters noted in the NC and Converter_post_ groups.

In summary, TEs appear to be involved in a profound re-organization of the functional architecture of the genome in LOAD (and probably other age-dependent diseases). Based on our analyses, DE TEs at specific Converter_pre_ timepoints appear to accurately identify those individuals that are at risk of *pheno*converting to LOAD. Two different analytical methodologies were used to define such biomarkers: using either (1) all the DE TEs identified between Converter_pre_ and NC or (2) a machine learning algorithm that makes use of a much reduced number of DE TEs, after a thorough control of entropy reduction. While it is not surprising that the whole set of DE TEs (1790 elements) can fully discriminate between the Converter_pre_ and Converter_post_ stages of LOAD development, it is interesting to note that only 8 TEs are required to discriminate subjects between Converter_pre_ and Converter_post_, with about 80% accuracy. Whether the latter result might be suggestive of a more biologically relevant set of TEs within the preclinical stage of LOAD, or is a consequence of the entropy reduction algorithm, remains unresolved, but it will require further elucidation.

Despite best efforts, our study’s analyses have limitations. First, our *pheno*converters providing evidence for a *TE storm* provide a relatively small sample size, with only 25 subjects transitioning from normal cognition to the symptomatic stages of LOAD during the 5-year study window. Although the study group is unique, with community-dwelling seniors providing longitudinal clinical data and specimens, allowing the assessment of preliminary clinical features for correlation with additional data, much larger sample sets are needed to confirm these preliminary findings and to allow a more in-depth and statistically robust analysis of the roles of TEs in LOAD. Second, a thorough understanding of the putative mechanistic role(s) played by TEs in the clinical transition to symptomatic stages of LOAD requires specific and detailed investigations at molecular and cellular levels. Potentially, each individual TE might have a specific contributory role in the evolution into manifest stages of LOAD. As such, the role(s) and ramification(s) for each TE should be fully assessed, a task that goes well-beyond the capabilities of a single lab, and requires a collaborative effort across multiple labs. Fortunately, a consistent and growing amount of new knowledge regarding the biology of TEs—as well as new methods to investigate putative and specific TE functions—are finally being developed, with this trend likely to continue into the future.

Next, it will be key to assess the functional role of TEs as regulatory elements in association with varying chromatin states. We strongly believe that developing epigenetic data, to complement the RNA expression profile of TEs, is critically relevant. To begin to address this key point—and without methylation/epigenetic data available at this time from our own samples—we have resorted to using the information provided by the Epigenome Roadmap consortium with a computational only approach. This analysis allowed us to also explore whether DE TEs identified in blood are showing signals of possible expression in different brain adult and fetal tissues.

Finally, the manifest LOAD diagnosis for our subjects is based on clinical and neuropsychological examinations, but not confirmed by any unequivocal objective measure. Our clinical diagnosis of LOAD, however, has additional support due to a clinical stability criterion, since the diagnoses of aMCI or LOAD remain stable for at least 2 years or worsen during follow-up examinations.

At present, our results do not have an immediate clinical translational applicability, but they only represent a proof of concept for the role that TEs can play in contributing to Alzheimer’s disease and possibly to neurodegeneration in general. A more extensive analytical framework is required to identify and characterize the specific, and time-dependent, roles portrayed by the varying TE classes, if not by individual TE elements. With increasing granularity in our knowledge base, it becomes evident that a detailed picture of the complex pathobiologic process will ultimately present itself, defining the actors involved and their roles in the mechanisms of action. Such increasing clarity will ultimately help to define and identify therapeutics and technologies to mitigate these conditions.

## Supplementary Information

Below is the link to the electronic supplementary material.

Supplementary file1 (PDF 1.26 MB)

Supplementary file2 (PDF 48.7 KB)

Supplementary file3 (PNG 154 KB)

Supplementary file4 (XLSX 215 KB)

Supplementary file5 (XLSX 64.9 KB)

Supplementary file6 (XLSX 124 KB)

Supplementary file7 (XLSX 86.7 KB)

Supplementary file8 (XLSX 343 KB)

Supplementary file9 (XLSX 105 KB)

## Data Availability

The raw data from RNA-sequencing are available from the corresponding author upon requests.

## References

[1] Ibanez L, Cruchaga C, Fernandez MV. Advances in genetic and molecular understanding of Alzheimer’s disease. Genes 2021, 12, 1247. 10.3390/genes12081247 10.3390/genes12081247 PMC8394321 34440421

[2] Frost B, Hemberg M, Lewis J, Feany MB (2014). Tau promotes neurodegeneration through global chromatin relaxation. Nat Neurosci.

[3] Colombo AR, Elias HK, Ramsingh G (2018). Senescence induction universally activates transposable element expression. Cell Cycle.

[4] Liu EY, Russ J, Cali CP, Phan JM, Amlie-Wolf A, Lee EB (2019). Loss of nuclear TDP-43 is associated with decondensation of LINE retrotransposons. Cell reports..

[5] Jonsson ME, Garza R, Johansson PA, Jakobsson J (2020). Transposable elements: a common feature of neurodevelopmental and neurodegenerative disorders. Trends Genet: TIG.

[6] Ochoa Thomas E, Zuniga G, Sun W, Frost B (2020). Awakening the dark side: retrotransposon activation in neurodegenerative disorders. Curr Opin Neurobiol.

[7] Hoyt SJ, Storer JM, Hartley GA, Grady PGS, Gershman A, de Lima LG, et al. From telomere to telomere: the transcriptional and epigenetic state of human repeat elements. Science 376, eabk3112 (2022). 10.1126/science.abk3112 10.1126/science.abk3112 PMC9301658 35357925

[8] Lander ES, Linton LM, Birren B, Nusbaum C, Zody MC, Baldwin J (2001). Initial sequencing and analysis of the human genome. Nature.

[9] de Koning APJ, Gu W, Castoe TA, Batzer MA, Pollock DD (2011). Repetitive elements may comprise over two-thirds of the human genome. PLoS Genet.

[10] Harpending HC, Batzer MA, Gurven M, Jorde LB, Rogers AR, Sherry ST (1998). Genetic traces of ancient demography. Proc Natl Acad Sci.

[11] Cordaux R, Batzer MA (2009). The impact of retrotransposons on human genome evolution. Nat Rev Genet.

[12] Berto S, Perdomo-Sabogal A, Gerighausen D, Qin J, Nowick K (2016). A Consensus network of gene regulatory factors in the human frontal lobe. Front Genet.

[13] Burns KH, Boeke JD (2012). Human transposon tectonics. Cell.

[14] Huang CR, Schneider AM, Lu Y, Niranjan T, Shen P, Robinson MA (2010). Mobile interspersed repeats are major structural variants in the human genome. Cell.

[15] Payer LM, Burns KH (2019). Transposable elements in human genetic disease. Nat Rev Genet.

[16] Hancks DC, Kazazian HH (2012). Active human retrotransposons: variation and disease. Curr Opin Genet Dev.

[17] Hancks DC, Kazazian HH (2016). Roles for retrotransposon insertions in human disease. Mob DNA.

[18] Solyom S, Kazazian HH (2012). Mobile elements in the human genome: implications for disease. Genome Med.

[19] Chuong EB, Elde NC, Feschotte C (2017). Regulatory activities of transposable elements: from conflicts to benefits. Nat Rev Genet.

[20] Guichard E, Peona V, Malagoli Tagliazucchi G, Abitante L, Jagoda E, Musella M (2018). Impact of non-LTR retrotransposons in the differentiation and evolution of anatomically modern humans. Mob DNA.

[21] Cosby RL, Chang N-C, Feschotte C (2019). Host-transposon interactions: conflict, cooperation, and cooption. Genes Dev.

[22] Goubert C, Zevallos NA, Feschotte C (2020). Contribution of unfixed transposable element insertions to human regulatory variation. Philos Trans R Soc B: Biol Sci.

[23] Cowley M, Oakey RJ (2013). Transposable elements re-wire and fine-tune the transcriptome. PLoS Genet.

[24] Glinsky GV (2016). Mechanistically distinct pathways of divergent regulatory DNA creation contribute to evolution of human-specific genomic regulatory networks driving phenotypic divergence of Homo sapiens. Genome Biol Evol.

[25] Garcia-Perez JL, Widmann TJ, Adams IR (2016). The impact of transposable elements on mammalian development. Development.

[26] Glinsky GV (2015). Transposable elements and DNA methylation create in embryonic stem cells human-specific regulatory sequences associated with distal enhancers and noncoding RNAs. Genome Biol Evol.

[27] Coufal NG, Garcia-Perez JL, Peng GE, Yeo GW, Mu Y, Lovci MT (2009). L1 retrotransposition in human neural progenitor cells. Nature.

[28] Hutchins AP, Pei D (2015). Transposable elements at the center of the crossroads between embryogenesis, embryonic stem cells, reprogramming, and long non-coding RNAs. Sci Bull (Beijing).

[29] Deniz O, Ahmed M, Todd CD, Rio-Machin A, Dawson MA, Branco MR (2020). Endogenous retroviruses are a source of enhancers with oncogenic potential in acute myeloid leukaemia. Nat Commun.

[30] Etchegaray E, Naville M, Volff JN, Haftek-Terreau Z. Transposable element-derived sequences in vertebrate development. Mob DNA. 2021;12(1):1. 10.1186/s13100-020-00229-5 10.1186/s13100-020-00229-5 PMC7786948 33407840

[31] Roller M, Stamper E, Villar D, Izuogu O, Martin F, Redmond AM (2021). LINE retrotransposons characterize mammalian tissue-specific and evolutionarily dynamic regulatory regions. Genome Biol.

[32] Cao X, Zhang Y, Payer LM, Lords H, Steranka JP, Burns KH (2020). Polymorphic mobile element insertions contribute to gene expression and alternative splicing in human tissues. Genome Biol.

[33] Cao Y, Chen G, Wu G, Zhang X, McDermott J, Chen X (2019). Widespread roles of enhancer-like transposable elements in cell identity and long-range genomic interactions. Genome Res.

[34] Glinsky GV (2018). Contribution of transposable elements and distal enhancers to evolution of human-specific features of interphase chromatin architecture in embryonic stem cells. Chromosome Res.

[35] Notwell JH, Chung T, Heavner W, Bejerano G (2015). A family of transposable elements co-opted into developmental enhancers in the mouse neocortex. Nat Commun.

[36] Nakanishi A, Kobayashi N, Suzuki-Hirano A, Nishihara H, Sasaki T, Hirakawa M, et al. A sine-derived element constitutes a unique modular enhancer for mammalian diencephalic Fgf8. PLoS ONE. 2012;7(8):e43785. 10.1371/journal.pone.0043785 10.1371/journal.pone.0043785 PMC3427154 22937095

[37] Emera D, Yin J, Reilly SK, Gockley J, Noonan JP (2016). Origin and evolution of developmental enhancers in the mammalian neocortex. Proc Natl Acad Sci USA.

[38] Ali A, Han K, Liang P. Role of transposable elements in gene regulation in the human genome. Life 2021, 11, 118. 10.3390/life11020118 10.3390/life11020118 PMC7913837 33557056

[39] Branco MR, Chuong EB (2020). Crossroads between transposons and gene regulation. Philos Trans R Soc B Biol Sci.

[40] Todd CD, Deniz Ö, Taylor D, Branco MR. Functional evaluation of transposable elements as enhancers in mouse embryonic and trophoblast stem cells. eLife 2019;8:e44344. 10.7554/eLife.44344 10.7554/eLife.44344 PMC6544436 31012843

[41] Mita P, Boeke JD (2016). How retrotransposons shape genome regulation. Curr Opin Genet Dev.

[42] Elbarbary RA, Lucas BA, Maquat LE (2016). Retrotransposons as regulators of gene expression. Science (New York, NY).

[43] Lanciano S, Cristofari G. Measuring and interpreting transposable element expression. Nat Rev Genet. 2020;21(12):721-36. 10.1038/s41576-020-0251-y 32576954

[44] Enriquez-Gasca R, Gould PA, Rowe HM. Host gene regulation by transposable elements: the new, the old and the ugly. Viruses. 2020;12(10):1089. 10.3390/v12101089 10.3390/v12101089 PMC7650545 32993145

[45] Carullo NVN, Phillips III RA, Simon RC, Soto SAR, Hinds JE, Salisbury AJ, et al. Enhancer RNAs predict enhancer-gene regulatory links and are critical for enhancer function in neuronal systems. Nucleic Acids Res. 2020;48(17):9550-70 10.1093/nar/gkaa671 PMC7515708 32810208

[46] Lewis MW, Li S, Franco HL (2019). Transcriptional control by enhancers and enhancer RNAs. Transcription.

[47] Cardiello JF, Sanchez GJ, Allen MA, Dowell RD (2020). Lessons from eRNAs: understanding transcriptional regulation through the lens of nascent RNAs. Transcription.

[48] Arnold PR, Wells AD, Li XC (2019). Diversity and emerging roles of enhancer RNA in regulation of gene expression and cell fate. Front Cell Dev Biol.

[49] Xing J, Wang H, Belancio VP, Cordaux R, Deininger PL, Batzer MA (2006). Emergence of primate genes by retrotransposon-mediated sequence transduction. Proc Natl Acad Sci U S A.

[50] Feschotte C (2008). Transposable elements and the evolution of regulatory networks. Nat Rev Genet.

[51] Wacholder AC, Carvunis A-R (2021). New genes from borrowed parts. Science.

[52] Cosby RL, Judd J, Zhang R, Zhong A, Garry N, Pritham EJ (2021). Recurrent evolution of vertebrate transcription factors by transposase capture. Science..

[53] Belancio VP, Hedges DJ, Deininger PL (2008). Mammalian non-LTR retrotransposons: For better or worse, in sickness and in health. Genome Res.

[54] Emera D, Casola C, Lynch VJ, Wildman DE, Agnew D, Wagner GP (2012). Convergent evolution of endometrial prolactin expression in primates, mice, and elephants through the independent recruitment of transposable elements. Mol Biol Evol.

[55] Emera D, Wagner GP (2012). Transformation of a transposon into a derived prolactin promoter with function during human pregnancy. Proc Natl Acad Sci USA.

[56] Hezroni H, Koppstein D, Schwartz MG, Avrutin A, Bartel DP, Ulitsky I (2015). Principles of long noncoding RNA evolution derived from direct comparison of transcriptomes in 17 species. Cell Rep.

[57] Su M, Han D, Boyd-Kirkup J, Yu X, Han J-DJ (2014). Evolution of Alu elements toward enhancers. Cell Rep.

[58] Lynch VJ, Nnamani MC, Kapusta A, Brayer K, Plaza SL, Mazur EC (2015). Ancient transposable elements transformed the uterine regulatory landscape and transcriptome during the evolution of mammalian pregnancy. Cell Rep.

[59] Babaian A, Mager DL (2016). Endogenous retroviral promoter exaptation in human cancer. Mob DNA.

[60] Deininger P (2011). Alu elements: know the SINEs. Genome Biol.

[61] Deininger P, Morales ME, White TB, Baddoo M, Hedges DJ, Servant G (2017). A comprehensive approach to expression of L1 loci. Nucleic Acids Res.

[62] Belancio VP, Roy-Engel AM, Pochampally RR, Deininger PL (2010). Somatic expression of LINE-1 elements in human tissues. Nucleic Acids Res.

[63] Belancio VP, Hedges DJ, Deininger PL (2006). LINE-1 RNA splicing and influences on mammalian gene expression. Nucleic Acids Res.

[64] Guffanti G, Bartlett A, Klengel T, Klengel C, Hunter R, Glinsky G (2018). Novel bioinformatics approach identifies transcriptional profiles of lineage-specific transposable elements at distinct loci in the human dorsolateral prefrontal cortex. Mol Biol Evol.

[65] Goodier JL, Kazazian HH (2008). Retrotransposons revisited: the restraint and rehabilitation of parasites. Cell.

[66] Guffanti G, Gaudi S, Fallon JH, Sobell J, Potkin SG, Pato C (2014). Transposable elements and psychiatric disorders. Am J Med Genet B Neuropsychiatr Genet.

[67] Evrony GD, Cai X, Lee E, Hills LB, Elhosary PC, Lehmann HS (2012). Single-neuron sequencing analysis of L1 retrotransposition and somatic mutation in the human brain. Cell.

[68] Poduri A, Evrony GD, Cai X, Walsh CA (2013). Somatic mutation, genomic variation, and neurological disease. Science.

[69] Erwin JA, Paquola AC, Singer T, Gallina I, Novotny M, Quayle C (2016). L1-associated genomic regions are deleted in somatic cells of the healthy human brain. Nat Neurosci.

[70] Macia A, Widmann TJ, Heras SR, Ayllon V, Sanchez L, Benkaddour-Boumzaouad M (2017). Engineered LINE-1 retrotransposition in nondividing human neurons. Genome Res.

[71] Marchetto MCN, Narvaiza I, Denli AM, Benner C, Lazzarini TA, Nathanson JL (2013). Differential L1 regulation in pluripotent stem cells of humans and apes. Nature.

[72] Muotri AR, Chu VT, Marchetto MC, Deng W, Moran JV, Gage FH (2005). Somatic mosaicism in neuronal precursor cells mediated by L1 retrotransposition. Nature.

[73] Muotri AR, Gage FH (2006). Generation of neuronal variability and complexity. Nature.

[74] Muotri AR, Marchetto MCN, Coufal NG, Oefner R, Yeo G, Nakashima K (2010). L1 retrotransposition in neurons is modulated by MeCP2. Nature.

[75] Muotri AR, Zhao C, Marchetto MCN, Gage FH (2009). Environmental influence on L1 retrotransposons in the adult hippocampus. Hippocampus.

[76] Liu N, Lee CH, Swigut T, Grow E, Gu B, Bassik MC (2018). Selective silencing of euchromatic L1s revealed by genome-wide screens for L1 regulators. Nature.

[77] Long HK, Prescott SL, Wysocka J (2016). Ever-changing landscapes: transcriptional enhancers in development and evolution. Cell.

[78] Prescott SL, Srinivasan R, Marchetto MC, Grishina I, Narvaiza I, Selleri L (2015). Enhancer divergence and cis-regulatory evolution in the human and chimp neural crest. Cell.

[79] Sundaram V, Wysocka J (2020). Transposable elements as a potent source of diverse cis-regulatory sequences in mammalian genomes. Philos Trans R Soc B Biol Sci.

[80] Subramanian RP, Wildschutte JH, Russo C, Coffin JM (2011). Identification, characterization, and comparative genomic distribution of the HERV-K (HML-2) group of human endogenous retroviruses. Retrovirology.

[81] Hegyi H (2013). GABBR1 has a HERV-W LTR in its regulatory region–a possible implication for schizophrenia. Biol Direct.

[82] Suntsova M, Garazha A, Ivanova A, Kaminsky D, Zhavoronkov A, Buzdin A (2015). Molecular functions of human endogenous retroviruses in health and disease. Cell Mol Life Sci.

[83] Suntsova M, Gogvadze EV, Salozhin S, Gaifullin N, Eroshkin F, Dmitriev SE (2013). Human-specific endogenous retroviral insert serves as an enhancer for the schizophrenia-linked gene PRODH. Proc Natl Acad Sci U S A.

[84] Perron H, Hamdani N, Faucard R, Lajnef M, Jamain S, Daban-Huard C (2012). Molecular characteristics of human endogenous retrovirus type-W in schizophrenia and bipolar disorder. Transl Psychiatry.

[85] Perron H, Germi R, Bernard C, Garcia-Montojo M, Deluen C, Farinelli L (2012). Human endogenous retrovirus type W envelope expression in blood and brain cells provides new insights into multiple sclerosis disease. Mult Scler.

[86] Tangsuwansri C, Saeliw T, Thongkorn S, Chonchaiya W, Suphapeetiporn K, Mutirangura A (2018). Investigation of epigenetic regulatory networks associated with autism spectrum disorder (ASD) by integrated global LINE-1 methylation and gene expression profiling analyses. PLoS ONE.

[87] Shpyleva S, Melnyk S, Pavliv O, Pogribny I, Jill JS (2018). Overexpression of LINE-1 retrotransposons in autism brain. Mol Neurobiol.

[88] Balestrieri E, Arpino C, Matteucci C, Sorrentino R, Pica F, Alessandrelli R (2012). HERVs expression in autism spectrum disorders. PLoS ONE.

[89] Zhao B, Wu Q, Ye AY, Guo J, Zheng X, Yang X (2019). Somatic LINE-1 retrotransposition in cortical neurons and non-brain tissues of Rett patients and healthy individuals. PLoS Genet.

[90] Marchetto MC, Carromeu C, Acab A, Yu D, Yeo GW, Mu Y (2010). A model for neural development and treatment of Rett syndrome using human induced pluripotent stem cells. Cell.

[91] Reilly MT, Faulkner GJ, Dubnau J, Ponomarev I, Gage FH (2013). The role of transposable elements in health and diseases of the central nervous system. J Neurosci.

[92] De Cecco M, Ito T, Petrashen AP, Elias AE, Skvir NJ, Criscione SW (2019). L1 drives IFN in senescent cells and promotes age-associated inflammation. Nature.

[93] LaRocca TJ, Cavalier AN, Wahl D. Repetitive elements as a transcriptomic marker of aging: evidence in multiple datasets and models. Aging Cell. 2020;19(7):e13167. 10.1111/acel.13167 PMC7412685 32500641

[94] Nevalainen T, Autio A, Mishra BH, Marttila S, Jylhä M, Hurme M (2018). Aging-associated patterns in the expression of human endogenous retroviruses. PLoS ONE.

[95] Wood JG, Helfand SL (2013). Chromatin structure and transposable elements in organismal aging. Front Genet.

[96] Maxwell PH (2016). What might retrotransposons teach us about aging?. Curr Genet.

[97] Maxwell PH, Burhans WC, Curcio MJ (2011). Retrotransposition is associated with genome instability during chronological aging. Proc Natl Acad Sci USA.

[98] Protasova MS, Gusev FE, Grigorenko AP, Kuznetsova IL, Rogaev EI, Andreeva TV (2017). Quantitative analysis of L1-retrotransposons in Alzheimer’s disease and aging. Biochemistry Biokhimiia.

[99] De Cecco M, Criscione SW, Peterson AL, Neretti N, Sedivy JM, Kreiling JA (2013). Transposable elements become active and mobile in the genomes of aging mammalian somatic tissues. Aging-Us.

[100] Sun W, Samimi H, Gamez M, Zare H, Frost B (2018). Pathogenic tau-induced piRNA depletion promotes neuronal death through transposable element dysregulation in neurodegenerative tauopathies. Nat Neurosci.

[101] Guo C, Jeong H-H, Hsieh Y-C, Klein H-U, Bennett DA, De Jager PL (2018). Tau activates transposable elements in Alzheimer’s disease. Cell Rep.

[102] De Cecco M, Criscione SW, Peterson AL, Neretti N, Sedivy JM, Kreiling JA (2013). Transposable elements become active and mobile in the genomes of aging mammalian somatic tissues. Aging (Albany NY).

[103] Dembny P, Newman AG, Singh M, Hinz M, Szczepek M, Kruger C, et al. Human endogenous retrovirus HERV-K(HML-2) RNA causes neurodegeneration through Toll-like receptors. JCI Insight. 2020;5(7):e131093. 10.1172/jci.insight.131093 10.1172/jci.insight.131093 PMC7205273 32271161

[104] Curzio DD, Gurm M, Turnbull M, Nadeau MJ, Meek B, Rempel JD, et al. Pro-inflammatory signaling upregulates a neurotoxic conotoxin-like protein encrypted within human endogenous retrovirus-K. Cells. 2020;9(7):1584. 10.3390/cells9071584 10.3390/cells9071584 PMC7407490 32629888

[105] Mapstone M, Cheema AK, Fiandaca MS, Zhong X, Mhyre TR, MacArthur LH (2014). Plasma phospholipids identify antecedent memory impairment in older adults. Nat Med.

[106] Fiandaca MS, Zhong X, Cheema AK, Orquiza MH, Chidambaram S, Tan MT (2015). Plasma 24-metabolite panel predicts preclinical transition to clinical stages of Alzheimer’s disease. Front Neurol.

[107] Leek JT. svaseq: removing batch effects and other unwanted noise from sequencing data. Nucleic Acids Res. 2014;42(21):e161 doi: 10.1093/nar/gku864 10.1093/nar/gku864 PMC4245966 25294822

[108] Haas BJ, Papanicolaou A, Yassour M, Grabherr M, Blood PD, Bowden J (2013). De novo transcript sequence reconstruction from RNA-seq using the Trinity platform for reference generation and analysis. Nat Protoc.

[109] Grabherr MG, Haas BJ, Yassour M, Levin JZ, Thompson DA, Amit I (2011). Full-length transcriptome assembly from RNA-Seq data without a reference genome. Nat Biotechnol.

[110] Pertea M, Kim D, Pertea GM, Leek JT, Salzberg SL (2016). Transcript-level expression analysis of RNA-seq experiments with HISAT. StringTie and Ballgown Nat Protoc.

[111] Bray NL, Pimentel H, Melsted P, Pachter L (2016). Near-optimal probabilistic RNA-seq quantification. Nat Biotechnol.

[112] Robinson MD, Oshlack A (2010). A scaling normalization method for differential expression analysis of RNA-seq data. Genome Biol.

[113] Robinson MD, McCarthy DJ, Smyth GK (2010). edgeR: a Bioconductor package for differential expression analysis of digital gene expression data. Bioinformatics.

[114] McCarthy DJ, Chen Y, Smyth GK (2012). Differential expression analysis of multifactor RNA-Seq experiments with respect to biological variation. Nucleic Acids Res.

[115] Neph S, Kuehn MS, Reynolds AP, Haugen E, Thurman RE, Johnson AK (2012). BEDOPS: high-performance genomic feature operations. Bioinformatics.

[116] Hummel M, Edelmann D, Kopp-Schneider A (2017). Clustering of samples and variables with mixed-type data. PLoS ONE.

[117] Venables WN, Ripley BD. Modern applied statistics with S. New York: Springer; 2002.

[118] Qiu X, Mao Q, Tang Y, Wang L, Chawla R, Pliner HA (2017). Reversed graph embedding resolves complex single-cell trajectories. Nat Methods.

[119] Qiu X, Hill A, Packer J, Lin D, Ma YA, Trapnell C (2017). Single-cell mRNA quantification and differential analysis with Census. Nat Methods.

[120] Kursa MB, Rudnicki WR (2010). Feature selection with the Boruta package. J Stat Softw.

[121] Kuhn M (2008). Building predictive models in R using the Caret package. J Stat Softw.

[122] Wright MN, Ziegler AG (2017). ranger: A Fast Implementation of Random Forests for High Dimensional Data in C++ and R. J Stat Softw.

[123] Robin X, Turck N, Hainard A, Tiberti N, Lisacek F, Sanchez J (2011). pROC: an open-source package for R and S+ to analyze and compare ROC curves. BMC Bioinformatics.

[124] Trendafilov NT (2010). Stepwise estimation of common principal components. Comput Stat Data Anal.

[125] McLean CY, Bristor D, Hiller M, Clarke SL, Schaar BT, Lowe CB (2010). GREAT improves functional interpretation of cis-regulatory regions. Nat Biotechnol.

[126] Terreros MC, Alfonso-Sanchez MA, Novick GE, Luis JR, Lacau H, Lowery RK (2009). Insights on human evolution: an analysis of Alu insertion polymorphisms. J Hum Genet.

[127] Salem AH, Kilroy GE, Watkins WS, Jorde LB, Batzer MA (2003). Recently integrated Alu elements and human genomic diversity. Mol Biol Evol.

[128] Römer C, Singh M, Hurst LD, Izsvák Z (2017). How to tame an endogenous retrovirus: HERVH and the evolution of human pluripotency. Curr Opin Virol.

[129] Gemmell P, Hein J, Katzourakis A (2019). The exaptation of HERV-H: evolutionary analyses reveal the genomic features of highly transcribed elements. Front Immunol.

[130] Salem AH, Ray DA, Xing J, Callinan PA, Myers JS, Hedges DJ (2003). Alu elements and hominid phylogenetics. PNAS.

[131] Boissinot S, Chevret P, Furano AV (2000). L1 (LINE-1) retrotransposon evolution and amplification in recent human history. Mol Biol Evol.

[132] Khan H, Smit A, Boissinot S (2006). Molecular evolution and tempo of amplification of human LINE-1 retrotransposons since the origin of primates. Genome Res.

[133] Boissinot S, Furano AV (2001). Adaptive evolution in LINE-1 retrotransposons. Mol Biol Evol.

[134] Krings M, Salem AE, Bauer K, Geisert H, Malek AK, Chaix L (1999). mtDNA analysis of Nile River valley populations: a genetic corridor or a barrier to migration?. Am J Hum Genet.

[135] Kundaje A, Meuleman W, Ernst J, Bilenky M, Yen A, Heravi-Moussavi A (2015). Integrative analysis of 111 reference human epigenomes. Nature.

[136] Pehrsson EC, Choudhary MNK, Sundaram V, Wang T (2019). The epigenomic landscape of transposable elements across normal human development and anatomy. Nat Commun.

[137] Gireud-Goss M, Reyes S, Tewari R, Patrizz A, Howe MD, Kofler J (2020). The ubiquitin ligase UBE4B regulates amyloid precursor protein ubiquitination, endosomal trafficking, and amyloid beta42 generation and secretion. Mol Cell Neurosci.

[138] Wang ZH, Liu P, Liu X, Manfredsson FP, Sandoval IM, Yu SP (2017). Delta-secretase phosphorylation by SRPK2 enhances its enzymatic activity, provoking pathogenesis in Alzheimer’s disease. Mol Cell.

[139] Boutej H, Rahimian R, Thammisetty SS, Beland LC, Lalancette-Hebert M, Kriz J (2017). Diverging mRNA and protein networks in activated microglia reveal SRSF3 suppresses translation of highly upregulated innate immune transcripts. Cell Rep.

[140] Sanchez-Valle J, Tejero H, Ibanez K, Portero JL, Krallinger M, Al-Shahrour F (2017). A molecular hypothesis to explain direct and inverse co-morbidities between Alzheimer’s Disease, glioblastoma and lung cancer. Sci Rep.

[141] Zheng Q, Li G, Wang S, Zhou Y, Liu K, Gao Y (2021). Trisomy 21–induced dysregulation of microglial homeostasis in Alzheimer’s brains is mediated by USP25. Sci Adv.

[142] Soleimani Zakeri NS, Pashazadeh S, MotieGhader H (2020). Gene biomarker discovery at different stages of Alzheimer using gene co-expression network approach. Sci Rep.

[143] de Yebra L, Adroer R, de Gregorio-Rocasolano N, Blesa R, Trullas R, Mahy N (2004). Reduced KIAA0471 mRNA expression in Alzheimer’s patients: a new candidate gene product linked to the disease?. Hum Mol Genet.

[144] Castillo E, Leon J, Mazzei G, Abolhassani N, Haruyama N, Saito T (2017). Comparative profiling of cortical gene expression in Alzheimer’s disease patients and mouse models demonstrates a link between amyloidosis and neuroinflammation. Sci Rep.

[145] Cai CC, Langfelder P, Fuller TF, Oldham MC, Luo R, van den Berg LH, et al. Is human blood a good surrogate for brain tissue in transcriptional studies? BMC Genomics. 2010;11:589. http://www.biomedcentral.com/1471-2164/11/589 10.1186/1471-2164-11-589 PMC3091510 20961428

[146] Edgar RD, Jones MJ, Meaney MJ, Turecki G, Kobor MS (2017). BECon: a tool for interpreting DNA methylation findings from blood in the context of brain. Transl Psychiatry.

[147] Sedivy JM, Kreiling JA, Neretti N, De Cecco M, Criscione SW, Hofmann JW (2013). Death by transposition - the enemy within?. BioEssays.

[148] Sjostedt E, Zhong W, Fagerberg L, Karlsson M, Mitsios N, Adori C, et al. An atlas of the protein-coding genes in the human, pig, and mouse brain. Science. 2020;367(6482):eaay5947. 10.1126/science.aay5947 10.1126/science.aay5947 32139519

[149] Bushman DM, Kaeser GE, Siddoway B, Westra JW, Rivera RR, Rehen SK, et al. Genomic mosaicism with increased amyloid precursor protein (APP) gene copy number in single neurons from sporadic Alzheimer’s disease brains. Elife. 2015;4:e05116. 10.7554/eLife.05116 10.7554/eLife.05116 PMC4337608 25650802

[150] Chai G, Gleeson JG (2018). A mosaic mutation mechanism in the brain. Nature.

[151] Lee MH, Siddoway B, Kaeser GE, Segota I, Rivera R, Romanow WJ (2018). Somatic APP gene recombination in Alzheimer’s disease and normal neurons. Nature.

[152] Kim J, Zhao B, Huang AY, Miller MB, Lodato MA, Walsh CA (2020). APP gene copy number changes reflect exogenous contamination. Nature.

[153] Watkins WS, Feusier JE, Thomas J, Goubert C, Mallick S, Jorde LB (2020). The Simons Genome Diversity Project: a global analysis of mobile element diversity. Genome Biol Evol.

[154] Feusier J, Watkins WS, Thomas J, Farrell A, Witherspoon DJ, Baird L (2019). Pedigree-based estimation of human mobile element retrotransposition rates. Genome Res.

[155] Beck CR, Garcia-Perez JL, Badge RM, Moran JV (2011). LINE-1 elements in structural variation and disease. Annu Rev Genomics Hum Genet.

[156] Beck CR, Collier P, Macfarlane C, Malig M, Kidd JM, Eichler EE (2010). LINE-1 retrotransposition activity in human genomes. Cell.

[157] Ueberham U, Arendt T (2020). Genomic indexing by somatic gene recombination of mRNA/ncRNA - does it play a role in genomic mosaicism, memory formation, and Alzheimer’s disease?. Front Genet.

[158] Faulkner GJ, Garcia-Perez JL (2017). L1 mosaicism in mammals: extent, effects, and evolution. Trends Genet: TIG.

[159] Faulkner GJ, Kimura Y, Daub CO, Wani S, Plessy C, Irvine KM (2009). The regulated retrotransposon transcriptome of mammalian cells. Nat Genet.

[160] Ecco G, Cassano M, Kauzlaric A, Duc J, Coluccio A, Offner S (2016). Transposable elements and their KRAB-ZFP controllers regulate gene expression in adult tissues. Dev Cell.

[161] Lockstone HE, Harris LW, Swatton JE, Wayland MT, Holland AJ, Bahn S (2007). Gene expression profiling in the adult Down syndrome brain. Genomics.

[162] Suk TR, Rousseaux MWC (2020). The role of TDP-43 mislocalization in amyotrophic lateral sclerosis. Mol Neurodegener.

[163] Neumann M, Sampathu DM, Kwong LK, Truax AC, Micsenyi MC, Chou TT (2006). Ubiquitinated TDP-43 in frontotemporal lobar degeneration and amyotrophic lateral sclerosis. Science.

[164] Tziortzouda P, Van Den Bosch L, Hirth F (2021). Triad of TDP43 control in neurodegeneration: autoregulation, localization and aggregation. Nat Rev Neurosci.

[165] Li W, Jin Y, Prazak L, Hammell M, Dubnau J (2012). Transposable elements in TDP-43-mediated neurodegenerative disorders. PLoS ONE.

[166] Santoni FA, Guerra J, Luban J (2012). HERV-H RNA is abundant in human embryonic stem cells and a precise marker for pluripotency. Retrovirology.

[167] Grow EJ, Flynn RA, Chavez SL, Bayless NL, Wossidlo M, Wesche DJ (2015). Intrinsic retroviral reactivation in human preimplantation embryos and pluripotent cells. Nature.

[168] Wang J, Xie G, Singh M, Ghanbarian AT, Raskó T, Szvetnik A (2014). Primate-specific endogenous retrovirus-driven transcription defines naive-like stem cells. Nature.

[169] Lu X, Sachs F, Ramsay L, Jacques PE, Goke J, Bourque G (2014). The retrovirus HERVH is a long noncoding RNA required for human embryonic stem cell identity. Nat Struct Mol Biol.

[170] Reilly SK, Yin J, Ayoub AE, Emera D, Leng J, Cotney J (2015). Evolutionary changes in promoter and enhancer activity during human corticogenesis. Science.

[171] Stearrett N, Dawson T, Rahnavard A, Bachali P, Bendall ML, Zeng C (2021). Expression of human endogenous retroviruses in systemic lupus erythematosus: multiomic integration with gene expression. Front Immunol.

[172] Sankowski R, Strohl JJ, Huerta TS, Nasiri E, Mazzarello AN, D’Abramo C (2019). Endogenous retroviruses are associated with hippocampus-based memory impairment. Proc Natl Acad Sci U S A.

