# Supplementary material for: A retrotransposon storm marks clinical phenoconversion to late-onset Alzheimer’s disease
Source: GeroScience. 2022 May 19;44(3):1525–50. doi: 10.1007/s11357-022-00580-w (PMC9213607; doi:10.1007/s11357-022-00580-w)
Supplement: Supplementary file 1 — Supplementary file1 (PDF 1.26 MB) [file 11357_2022_580_MOESM1_ESM.pdf]

Supplementary Material for

# A Retro-transposons storm marks clinical phenoconversion to late-onset Alzheimer's Disease.

Fabio Macciardi[1#], Maria Giulia Bacalini[2], Ricardo Miramontes[3], Alessio Boattini[4], Cristian Taccioli[5], Giorgia Modenini[4], Rond Malhas[3], Laura Anderlucci[6], Yuriy Gusev[7], Thomas J. Gross[3], Robert M. Padilla[3], Massimo S Fiandaca[3], Elizabeth Head[8], Guia Guffanti[9], Howard J. Federoff[3], Mark Mapstone [3]

This PDF file includes:

Supplementary Methods: Shannon entropy
Supplementary Table 1
Supplementary File 1
Supplementary Figure 1
Supplementary Figure 3

## Shannon Entropy

The concept of entropy was introduced in the early 19th century by Rudolf Julius Emanuel Clausius. It represents a characteristic quantity of the state of a physical system capable of expressing the ability of the system itself to be able to proceed to spontaneous transformations and, consequently, the loss of ability to do work when such transformations occur. In simplified terms, the value of entropy increases when the system undergoes spontaneous variations and therefore loses part of its ability to undergo such variations and perform work. In 1872 Ludwig Boltzmann generalized this concept through the study of statistical mechanics by defining entropy as the degree of disorder of a system. In 1948 Claude Elwood Shannon equated the degree of inaccuracy of a message with disorder. For Shannon, in fact, the entropy of information was the degree of complexity of a message that represents the minimum average number of symbols necessary for the encoding of the message itself. The maximum value for Shannon entropy is two, whereas the minimum is  zero. In our analysis we decided to use Shannon entropy (Entropy R package retrieved from https://cran.r-project.org/web/packages/entropy) in order to remove those TEs with no significant information (H<=1). Thirty-thousands up to ~600M were selected after applying the aforementioned cut-off.

Supplementary Table 1

| A. Pre vs Post | | | | | | | | | | |
|---|---|---|---|---|---|---|---|---|---|---|
| | | | | | | | evolutionary recent TEs | | | |
| | mapped_transcripts | no. | length | std | unique_TE | | L1HS / L1P* | HERVK / LTR5Hs | AluY* | SVA |
| | **LINE** | 138,199 | 385 | 262.1 | 94,372 | | 12,775 | | | |
| | **LTR** | 63,563 | 396 | 277.3 | 44,694 | | | 1,331 | | |
| | **SINE** | 196,654 | 332.3 | 1.4 | 180,058 | | | | 18,068 | |
| | **SVA** | 3,513 | 430 | 244 | 1,651 | | | | | 1,651 |
| | **DNA** | 22,582 | 343.1 | 220 | 17,662 | | | | | |
| | | | | | | | | | | |
| | Total = | 424,511 | 377.28 | 200.96 | 338,437 | | 33,825 | | | |
| | | | | | | | | | | |
| **B. Pre vs Normal** | | | | | | | | | | |
| | | | | | | | evolutionary recent TEs | | | |
| | mapped_transcripts | no. | length | std | unique_TE | | L1HS / L1P* | HERVK / LTR5Hs | AluY* | SVA |
| | **LINE** | 176,478 | 362 | 241.7 | 118,403 | | 28,377 | | | |
| | **LTR** | 73,888 | 374 | 260.6 | 49,810 | | | 2,741 | | |
| | **SINE** | 212,522 | 313.2 | 49.9 | 186,225 | | | | 19,336 | |
| | **SVA** | 4,047 | 420 | 245 | 1,811 | | | | | 1,811 |
| | **DNA** | 22,759 | 321.4 | 202.3 | 16,910 | | | | | |
| | | | | | | | | | | |
| | Total = | 489,694 | 358.12 | 199.9 | 373,159 | | 52,265 | | | |

*Supplementary Table 1 presents the details of the TE-mapping transcripts (their absolute number, length average and std, and how many unique TEs are mapped within each Class. Evolutionary recent TEs include L1HS and L1PA2/3/4, HERVK/LTR5Hs, AluY and SVA and account for 9.9% of all TEs in Converter$_{pre}$ vs Converter$_{post}$ and 14% of all TEs in Converter$_{pre}$ vs NC.*

Supplementary File 1

A. *Converter*$_{pre}$ *vs Converter*$_{post}$

| UP-REGULATED | | | | |
|---|---|---|---|---|
| | | OR | 95% CI | p-value |
| 1_TssA | | 0.57 | 0.15 - 1.46 | 0.340861544 |
| 2_TssAFlnk | | 0.67 | 0.29 - 1.32 | 0.307603347 |
| 3_TxFlnk | | 0.91 | 0.02 - 5.09 | 1 |
| 4_Tx | | 1 | 0.88 - 1.13 | 1 |
| 5_TxWk | | 1.41 | 1.27 - 1.56 | 3.80E-11 |
| 6_EnhG | | 1.49 | 0.99 - 2.15 | 0.039622727 |
| 7_Enh | | 1.6 | 1.24 - 2.03 | 0.000288721 |
| 8_ZNF_Rpts | | 1.7 | 1.07 - 2.57 | 0.018983822 |
| 9_Het | | 0.97 | 0.67 - 1.36 | 0.931819995 |
| 10_TssBiv | | 0 | 0 - 24.25 | 1 |
| 11_BivFlnk | | 0 | 0 - 15.69 | 1 |
| 12_EnhBiv | | 0 | 0 - 9.54 | 1 |
| 13_ReprPC | | 0.61 | 0.07 - 2.21 | 0.777938556 |
| 14_ReprPCWk | | 0.53 | 0.37 - 0.75 | 6.97E-05 |
| 15_Quies | | 0.84 | 0.75 - 0.94 | 0.002587397 |

| DOWN-REGULATED | | | | |
|---|---|---|---|---|
| | | OR | 95% CI | p-value |
| 1_TssA | | 3.66 | 0.99 - 9.51 | 0.025977778 |
| 2_TssAFlnk | | 1.06 | 0.13 - 3.88 | 0.713752222 |
| 3_TxFlnk | | 0 | 0 - 21.44 | 1 |
| 4_Tx | | 1.03 | 0.75 - 1.39 | 0.877770067 |
| 5_TxWk | | 0.94 | 0.72 - 1.24 | 0.688639541 |
| 6_EnhG | | 0.64 | 0.08 - 2.35 | 0.774808223 |
| 7_Enh | | 0.54 | 0.15 - 1.41 | 0.339695261 |
| 8_ZNF_Rpts | | 1.88 | 0.51 - 4.88 | 0.169770565 |
| 9_Het | | 1.84 | 0.87 - 3.46 | 0.077883272 |
| 10_TssBiv | | 0 | 0 - 155.35 | 1 |
| 11_BivFlnk | | 0 | 0 - 100.57 | 1 |
| 12_EnhBiv | | 0 | 0 - 61.12 | 1 |
| 13_ReprPC | | 0 | 0 - 7.21 | 1 |
| 14_ReprPCWk | | 0.99 | 0.47 - 1.85 | 1 |
| 15_Quies | | 1.21 | 0.92 - 1.58 | 0.168313405 |

B. *Converter$_{pre}$ vs NC*

**UP-REGULATED**

| | | OR | 95% CI | p-value |
|---|---|---|---|---|
| **1_TssA** | | 1.18 | 0.14 - 4.29 | 0.689375571 |
| **2_TssAFlnk** | | 1.04 | 0.21 - 3.07 | 0.767061205 |
| **3_TxFlnk** | | 0 | 0 - 13.61 | 1 |
| **4_Tx** | | 0.96 | 0.74 - 1.23 | 0.805331433 |
| **5_TxWk** | | 1.31 | 1.06 - 1.62 | 0.01003909 |
| **6_EnhG** | | 1.04 | 0.34 - 2.45 | 0.817658559 |
| **7_Enh** | | 1.17 | 0.62 - 2.03 | 0.540749816 |
| **8_ZNF_Rpts** | | 0.93 | 0.19 - 2.74 | 1 |
| **9_Het** | | 0.82 | 0.33 - 1.71 | 0.729511639 |
| **10_TssBiv** | | 0 | 0 - 98.45 | 1 |
| **11_BivFlnk** | | 0 | 0 - 67.98 | 1 |
| **12_EnhBiv** | | 0 | 0 - 36.34 | 1 |
| **13_ReprPC** | | 0 | 0 - 4.56 | 1 |
| **14_ReprPCWk** | | 0.56 | 0.25 - 1.07 | 0.092656096 |
| **15_Quies** | | 1.07 | 0.86 - 1.33 | 0.543753359 |

**DOWN-REGULATED**

| | | OR | 95% CI | p-value |
|---|---|---|---|---|
| **1_TssA** | | 0 | 0 - 7.04 | 1 |
| **2_TssAFlnk** | | 1.11 | 0.03 - 6.33 | 0.594611785 |
| **3_TxFlnk** | | 0 | 0 - 44.16 | 1 |
| **4_Tx** | | 0.35 | 0.17 - 0.65 | 0.000234775 |
| **5_TxWk** | | 1.06 | 0.72 - 1.57 | 0.77354514 |
| **6_EnhG** | | 0 | 0 - 2.46 | 0.41130044 |
| **7_Enh** | | 0 | 0 - 1.05 | 0.053122746 |
| **8_ZNF_Rpts** | | 3.04 | 0.62 - 9.11 | 0.080579219 |
| **9_Het** | | 1.93 | 0.61 - 4.64 | 0.195744595 |
| **10_TssBiv** | | 0 | 0 - 316.55 | 1 |
| **11_BivFlnk** | | 58.67 | 1.45 - 342.21 | 0.017275135 |
| **12_EnhBiv** | | 0 | 0 - 117.65 | 1 |
| **13_ReprPC** | | 0 | 0 - 14.8 | 1 |
| **14_ReprPCWk** | | 1.23 | 0.44 - 2.77 | 0.640098929 |
| **15_Quies** | | 2.11 | 1.44 - 3.1 | 6.59E-05 |

Supplementary Figure 1

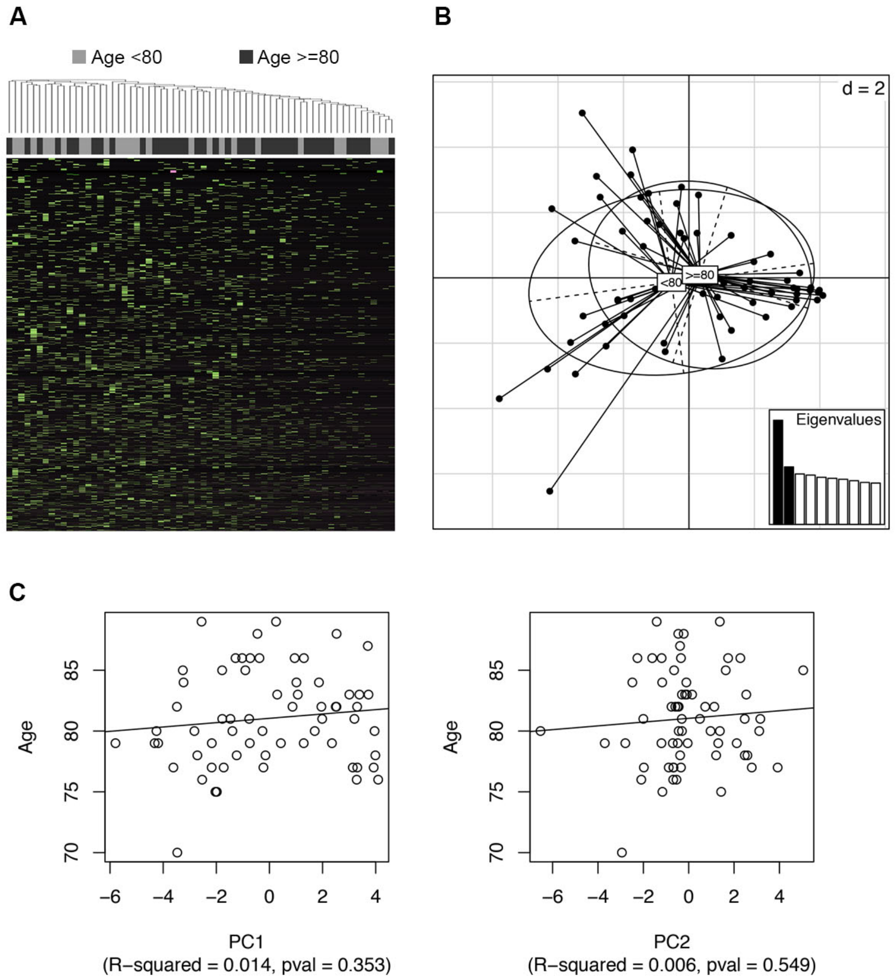


*Supplementary Figure 1A shows the absence of any clustering in the heatmap that compare "young" with "old" normal controls. Supplementary Figure 1B shows the results of a PCA analysis for these 2 groups considering the 1,790 DE TEs presenting an equal dispersion of subjects across PC1 and PC2. Supplementary Figure 1C shows a not significant result for age and both PC1 and PC2 in a linear model.*

Supplementary Figure 3

A.

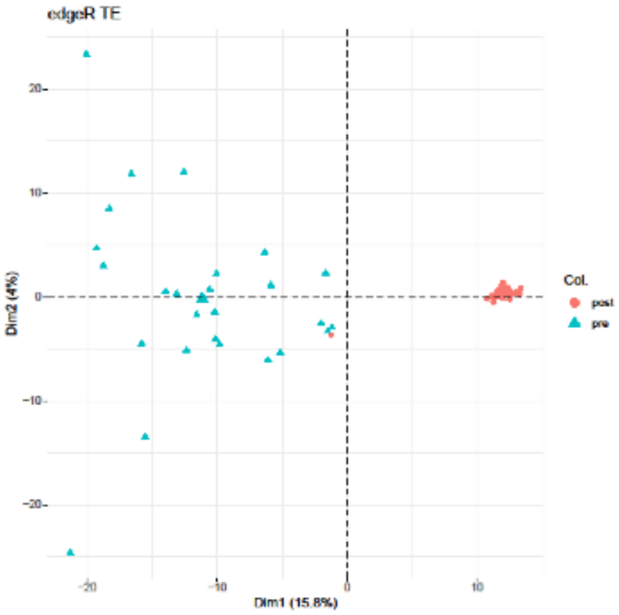


B.

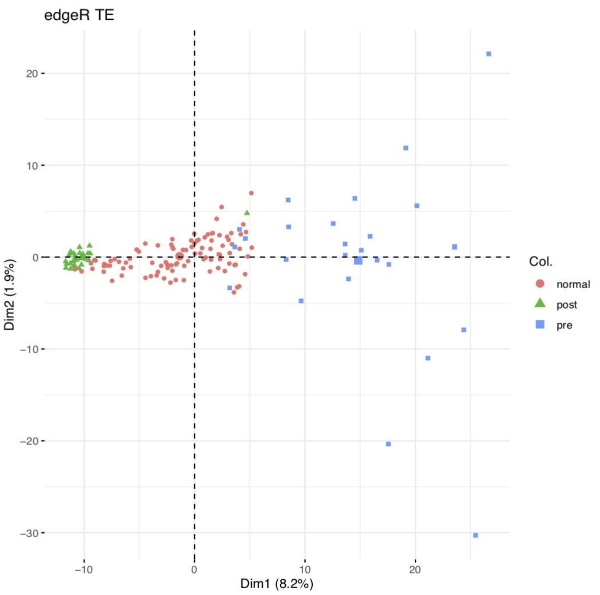


*Supplementary Figure 3. A. PCA of edgeR significant TEs using only Converter$_{pre}$ and Converter$_{post}$ individuals B. PCA of edgeR significant TEs using Converter$_{pre}$, Converter$_{post}$, and NC individuals.*
