# Supplementary material for: A retrotransposon storm marks clinical phenoconversion to late-onset Alzheimer’s disease
Source: GeroScience. 2022 May 19;44(3):1525–50. doi: 10.1007/s11357-022-00580-w (PMC9213607; doi:10.1007/s11357-022-00580-w)
Supplement: Supplementary file 2 — Supplementary file2 (PDF 48.7 KB) [file 11357_2022_580_MOESM2_ESM.pdf]

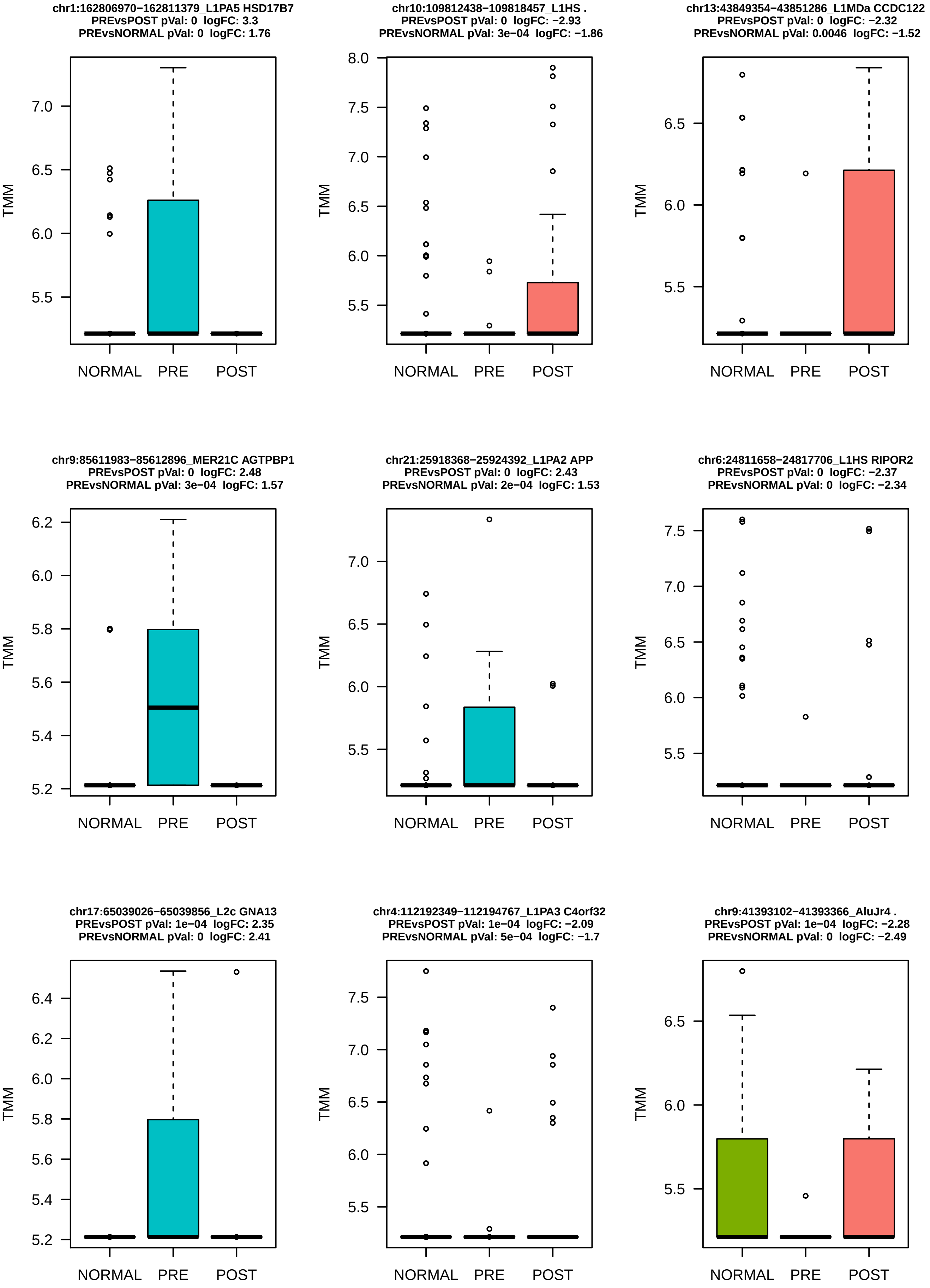
chr1:162806970−162811379_L1PA5 HSD17B7
PREvsPOST pVal: 0 logFC: 3.3
PREvsNORMAL pVal: 0 logFC: 1.76
TMM
5.5
6.0
6.5
7.0
NORMAL
PRE
POST
chr10:109812438−109818457_L1HS .
PREvsPOST pVal: 0 logFC: −2.93
PREvsNORMAL pVal: 3e−04 logFC: −1.86
TMM
5.5
6.0
6.5
7.0
7.5
8.0
NORMAL
PRE
POST
chr13:43849354−43851286_L1MDa CCDC122
PREvsPOST pVal: 0 logFC: −2.32
PREvsNORMAL pVal: 0.0046 logFC: −1.52
TMM
5.5
6.0
6.5
NORMAL
PRE
POST
chr9:85611983−85612896_MER21C AGTPBP1
PREvsPOST pVal: 0 logFC: 2.48
PREvsNORMAL pVal: 3e−04 logFC: 1.57
TMM
5.2
5.4
5.6
5.8
6.0
6.2
NORMAL
PRE
POST
chr21:25918368−25924392_L1PA2 APP
PREvsPOST pVal: 0 logFC: 2.43
PREvsNORMAL pVal: 2e−04 logFC: 1.53
TMM
5.5
6.0
6.5
7.0
NORMAL
PRE
POST
chr6:24811658−24817706_L1HS RIPOR2
PREvsPOST pVal: 0 logFC: −2.37
PREvsNORMAL pVal: 0 logFC: −2.34
TMM
5.5
6.0
6.5
7.0
7.5
NORMAL
PRE
POST
chr17:65039026−65039856_L2c GNA13
PREvsPOST pVal: 1e−04 logFC: 2.35
PREvsNORMAL pVal: 0 logFC: 2.41
TMM
5.2
5.4
5.6
5.8
6.0
6.2
6.4
NORMAL
PRE
POST
chr4:112192349−112194767_L1PA3 C4orf32
PREvsPOST pVal: 1e−04 logFC: −2.09
PREvsNORMAL pVal: 5e−04 logFC: −1.7
TMM
5.5
6.0
6.5
7.0
7.5
NORMAL
PRE
POST
chr9:41393102−41393366_AluJr4 .
PREvsPOST pVal: 1e−04 logFC: −2.28
PREvsNORMAL pVal: 0 logFC: −2.49
TMM
5.5
6.0
6.5
NORMAL
PRE
POST

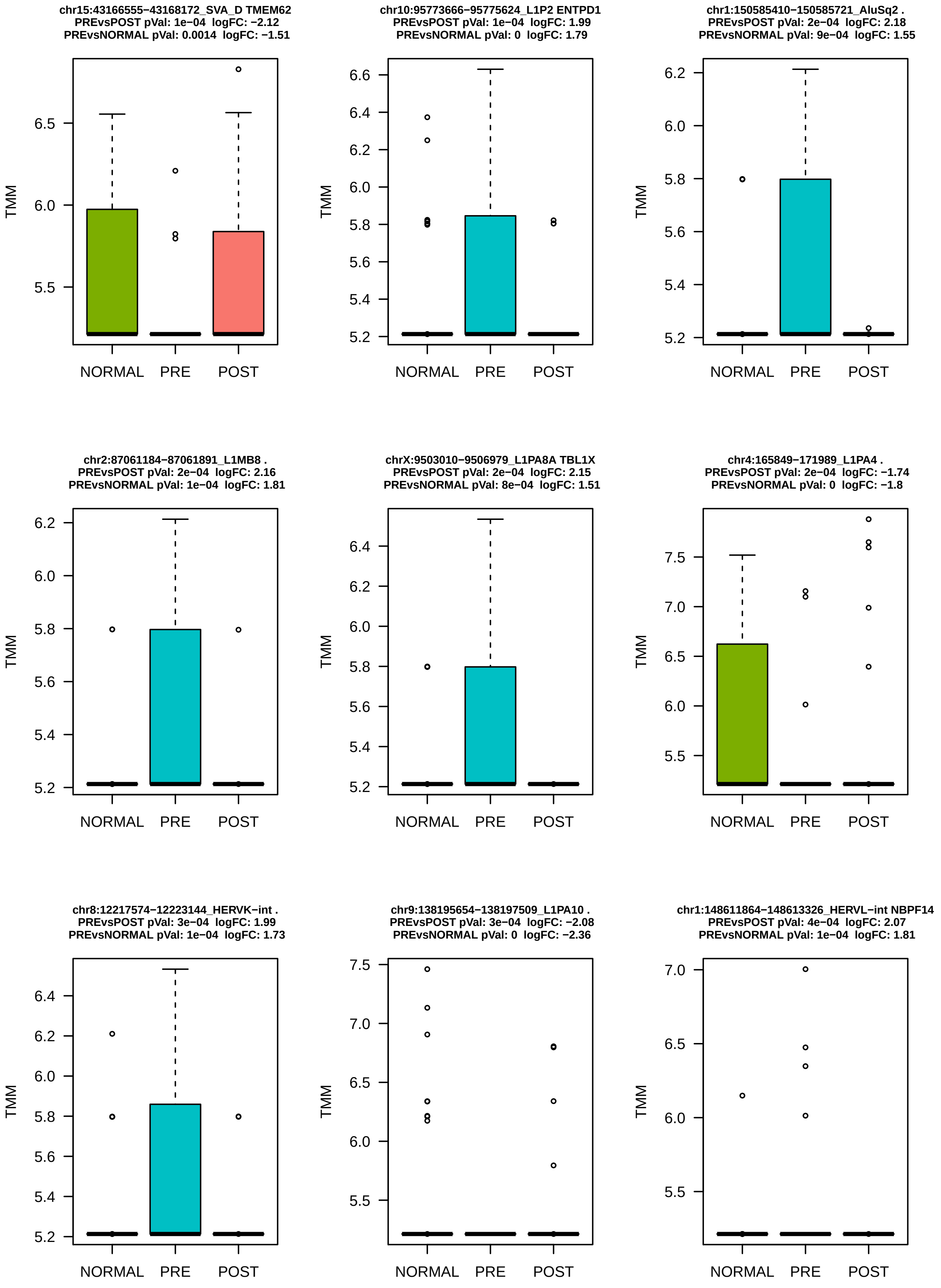
chr15:43166555−43168172_SVA_D TMEM62
PREvsPOST pVal: 1e−04 logFC: −2.12
PREvsNORMAL pVal: 0.0014 logFC: −1.51
chr10:95773666−95775624_L1P2 ENTPD1
PREvsPOST pVal: 1e−04 logFC: 1.99
PREvsNORMAL pVal: 0 logFC: 1.79
chr1:150585410−150585721_AluSq2 .
PREvsPOST pVal: 2e−04 logFC: 2.18
PREvsNORMAL pVal: 9e−04 logFC: 1.55
chr2:87061184−87061891_L1MB8 .
PREvsPOST pVal: 2e−04 logFC: 2.16
PREvsNORMAL pVal: 1e−04 logFC: 1.81
chrX:9503010−9506979_L1PA8A TBL1X
PREvsPOST pVal: 2e−04 logFC: 2.15
PREvsNORMAL pVal: 8e−04 logFC: 1.51
chr4:165849−171989_L1PA4 .
PREvsPOST pVal: 2e−04 logFC: −1.74
PREvsNORMAL pVal: 0 logFC: −1.8
chr8:12217574−12223144_HERVK−int .
PREvsPOST pVal: 3e−04 logFC: 1.99
PREvsNORMAL pVal: 1e−04 logFC: 1.73
chr9:138195654−138197509_L1PA10 .
PREvsPOST pVal: 3e−04 logFC: −2.08
PREvsNORMAL pVal: 0 logFC: −2.36
chr1:148611864−148613326_HERVL−int NBPF14
PREvsPOST pVal: 4e−04 logFC: 2.07
PREvsNORMAL pVal: 1e−04 logFC: 1.81
TMM
NORMAL
PRE
POST

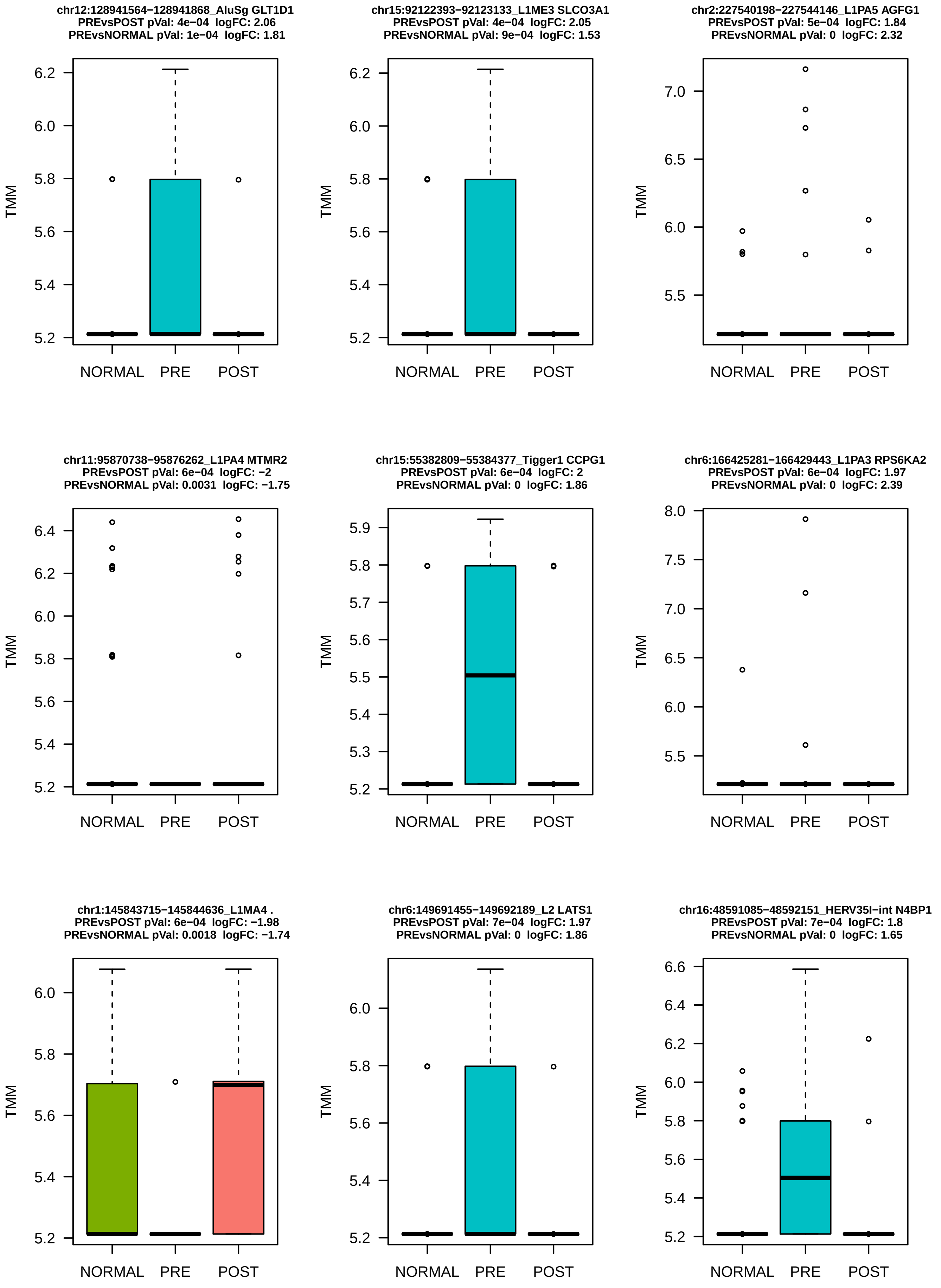
chr12:128941564−128941868_AluSg GLT1D1
PREvsPOST pVal: 4e−04 logFC: 2.06
PREvsNORMAL pVal: 1e−04 logFC: 1.81
chr15:92122393−92123133_L1ME3 SLCO3A1
PREvsPOST pVal: 4e−04 logFC: 2.05
PREvsNORMAL pVal: 9e−04 logFC: 1.53
chr2:227540198−227544146_L1PA5 AGFG1
PREvsPOST pVal: 5e−04 logFC: 1.84
PREvsNORMAL pVal: 0 logFC: 2.32
chr11:95870738−95876262_L1PA4 MTMR2
PREvsPOST pVal: 6e−04 logFC: −2
PREvsNORMAL pVal: 0.0031 logFC: −1.75
chr15:55382809−55384377_Tigger1 CCPG1
PREvsPOST pVal: 6e−04 logFC: 2
PREvsNORMAL pVal: 0 logFC: 1.86
chr6:166425281−166429443_L1PA3 RPS6KA2
PREvsPOST pVal: 6e−04 logFC: 1.97
PREvsNORMAL pVal: 0 logFC: 2.39
chr1:145843715−145844636_L1MA4 .
PREvsPOST pVal: 6e−04 logFC: −1.98
PREvsNORMAL pVal: 0.0018 logFC: −1.74
chr6:149691455−149692189_L2 LATS1
PREvsPOST pVal: 7e−04 logFC: 1.97
PREvsNORMAL pVal: 0 logFC: 1.86
chr16:48591085−48592151_HERV35I−int N4BP1
PREvsPOST pVal: 7e−04 logFC: 1.8
PREvsNORMAL pVal: 0 logFC: 1.65
TMM
NORMAL
PRE
POST

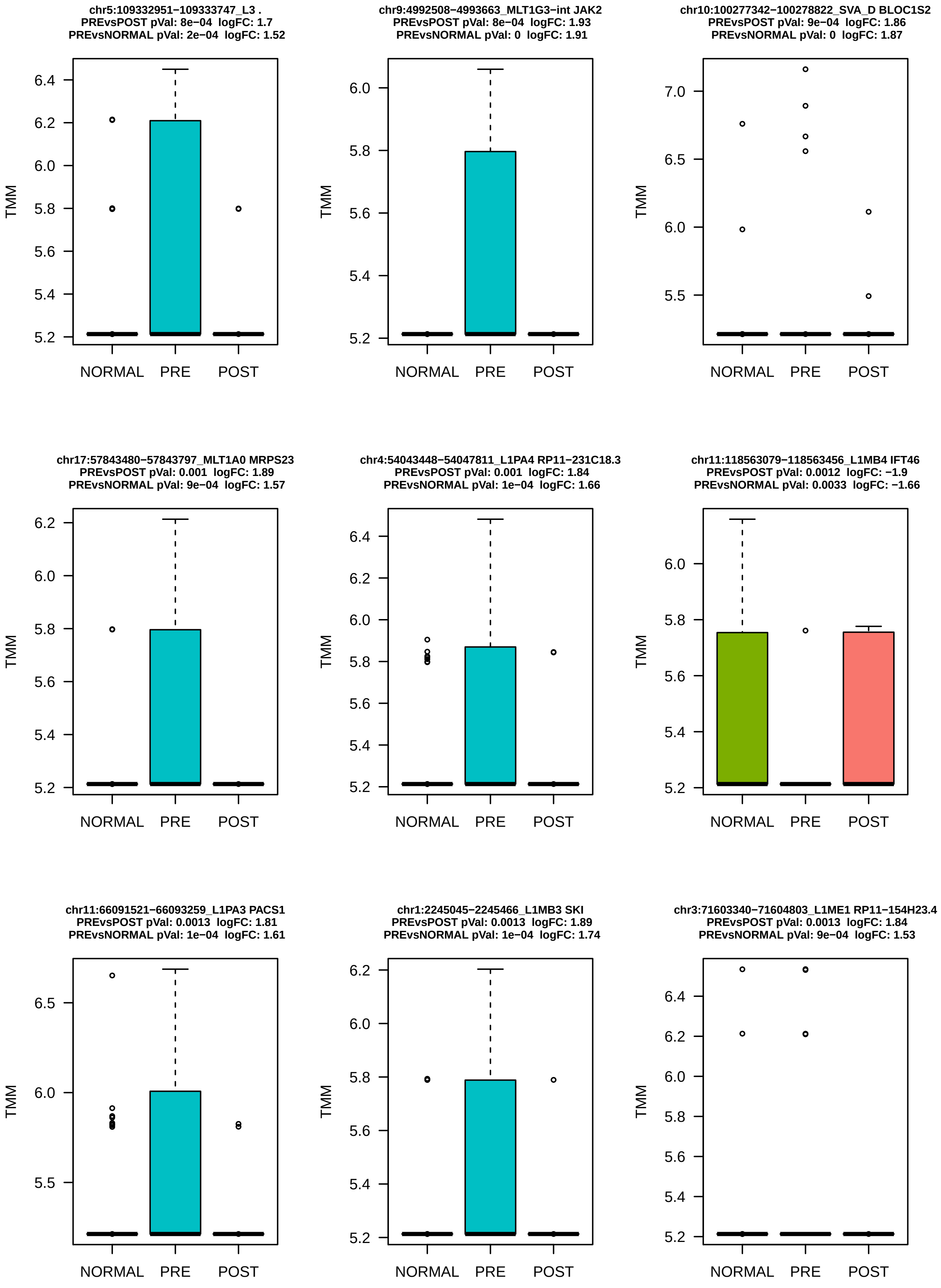

chr5:109332951−109333747_L3 .
PREvsPOST pVal: 8e−04 logFC: 1.7
PREvsNORMAL pVal: 2e−04 logFC: 1.52
chr9:4992508−4993663_MLT1G3−int JAK2
PREvsPOST pVal: 8e−04 logFC: 1.93
PREvsNORMAL pVal: 0 logFC: 1.91
chr10:100277342−100278822_SVA_D BLOC1S2
PREvsPOST pVal: 9e−04 logFC: 1.86
PREvsNORMAL pVal: 0 logFC: 1.87
chr17:57843480−57843797_MLT1A0 MRPS23
PREvsPOST pVal: 0.001 logFC: 1.89
PREvsNORMAL pVal: 9e−04 logFC: 1.57
chr4:54043448−54047811_L1PA4 RP11−231C18.3
PREvsPOST pVal: 0.001 logFC: 1.84
PREvsNORMAL pVal: 1e−04 logFC: 1.66
chr11:118563079−118563456_L1MB4 IFT46
PREvsPOST pVal: 0.0012 logFC: −1.9
PREvsNORMAL pVal: 0.0033 logFC: −1.66
chr11:66091521−66093259_L1PA3 PACS1
PREvsPOST pVal: 0.0013 logFC: 1.81
PREvsNORMAL pVal: 1e−04 logFC: 1.61
chr1:2245045−2245466_L1MB3 SKI
PREvsPOST pVal: 0.0013 logFC: 1.89
PREvsNORMAL pVal: 1e−04 logFC: 1.74
chr3:71603340−71604803_L1ME1 RP11−154H23.4
PREvsPOST pVal: 0.0013 logFC: 1.84
PREvsNORMAL pVal: 9e−04 logFC: 1.53
TMM
NORMAL
PRE
POST

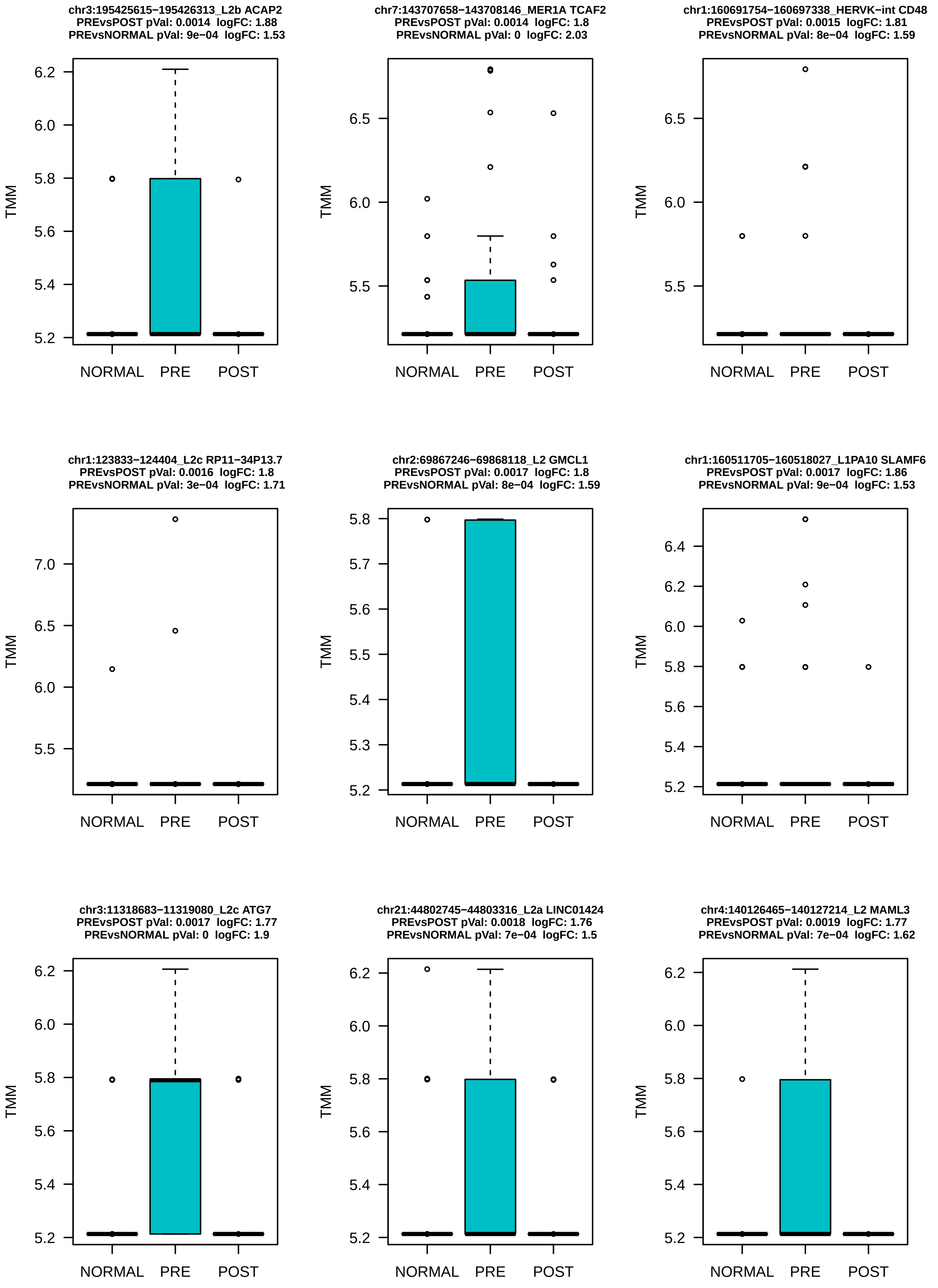
chr3:195425615−195426313_L2b ACAP2
PREvsPOST pVal: 0.0014 logFC: 1.88
PREvsNORMAL pVal: 9e−04 logFC: 1.53
chr7:143707658−143708146_MER1A TCAF2
PREvsPOST pVal: 0.0014 logFC: 1.8
PREvsNORMAL pVal: 0 logFC: 2.03
chr1:160691754−160697338_HERVK−int CD48
PREvsPOST pVal: 0.0015 logFC: 1.81
PREvsNORMAL pVal: 8e−04 logFC: 1.59
chr1:123833−124404_L2c RP11−34P13.7
PREvsPOST pVal: 0.0016 logFC: 1.8
PREvsNORMAL pVal: 3e−04 logFC: 1.71
chr2:69867246−69868118_L2 GMCL1
PREvsPOST pVal: 0.0017 logFC: 1.8
PREvsNORMAL pVal: 8e−04 logFC: 1.59
chr1:160511705−160518027_L1PA10 SLAMF6
PREvsPOST pVal: 0.0017 logFC: 1.86
PREvsNORMAL pVal: 9e−04 logFC: 1.53
chr3:11318683−11319080_L2c ATG7
PREvsPOST pVal: 0.0017 logFC: 1.77
PREvsNORMAL pVal: 0 logFC: 1.9
chr21:44802745−44803316_L2a LINC01424
PREvsPOST pVal: 0.0018 logFC: 1.76
PREvsNORMAL pVal: 7e−04 logFC: 1.5
chr4:140126465−140127214_L2 MAML3
PREvsPOST pVal: 0.0019 logFC: 1.77
PREvsNORMAL pVal: 7e−04 logFC: 1.62
TMM
NORMAL
PRE
POST

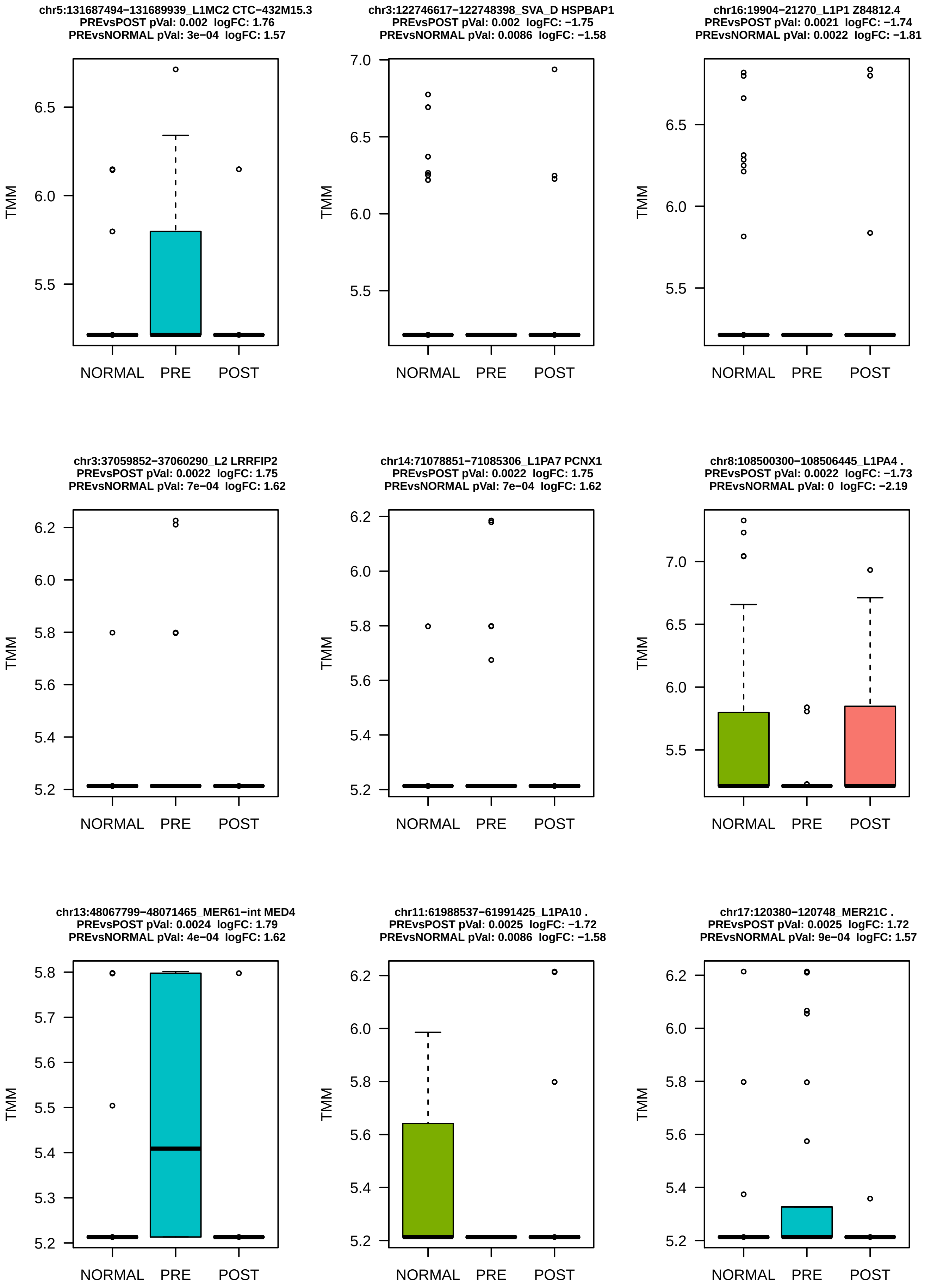
chr5:131687494−131689939_L1MC2 CTC−432M15.3
PREvsPOST pVal: 0.002 logFC: 1.76
PREvsNORMAL pVal: 3e−04 logFC: 1.57
chr3:122746617−122748398_SVA_D HSPBAP1
PREvsPOST pVal: 0.002 logFC: −1.75
PREvsNORMAL pVal: 0.0086 logFC: −1.58
chr16:19904−21270_L1P1 Z84812.4
PREvsPOST pVal: 0.0021 logFC: −1.74
PREvsNORMAL pVal: 0.0022 logFC: −1.81
chr3:37059852−37060290_L2 LRRFIP2
PREvsPOST pVal: 0.0022 logFC: 1.75
PREvsNORMAL pVal: 7e−04 logFC: 1.62
chr14:71078851−71085306_L1PA7 PCNX1
PREvsPOST pVal: 0.0022 logFC: 1.75
PREvsNORMAL pVal: 7e−04 logFC: 1.62
chr8:108500300−108506445_L1PA4 .
PREvsPOST pVal: 0.0022 logFC: −1.73
PREvsNORMAL pVal: 0 logFC: −2.19
chr13:48067799−48071465_MER61−int MED4
PREvsPOST pVal: 0.0024 logFC: 1.79
PREvsNORMAL pVal: 4e−04 logFC: 1.62
chr11:61988537−61991425_L1PA10 .
PREvsPOST pVal: 0.0025 logFC: −1.72
PREvsNORMAL pVal: 0.0086 logFC: −1.58
chr17:120380−120748_MER21C .
PREvsPOST pVal: 0.0025 logFC: 1.72
PREvsNORMAL pVal: 9e−04 logFC: 1.57
TMM
NORMAL
PRE
POST

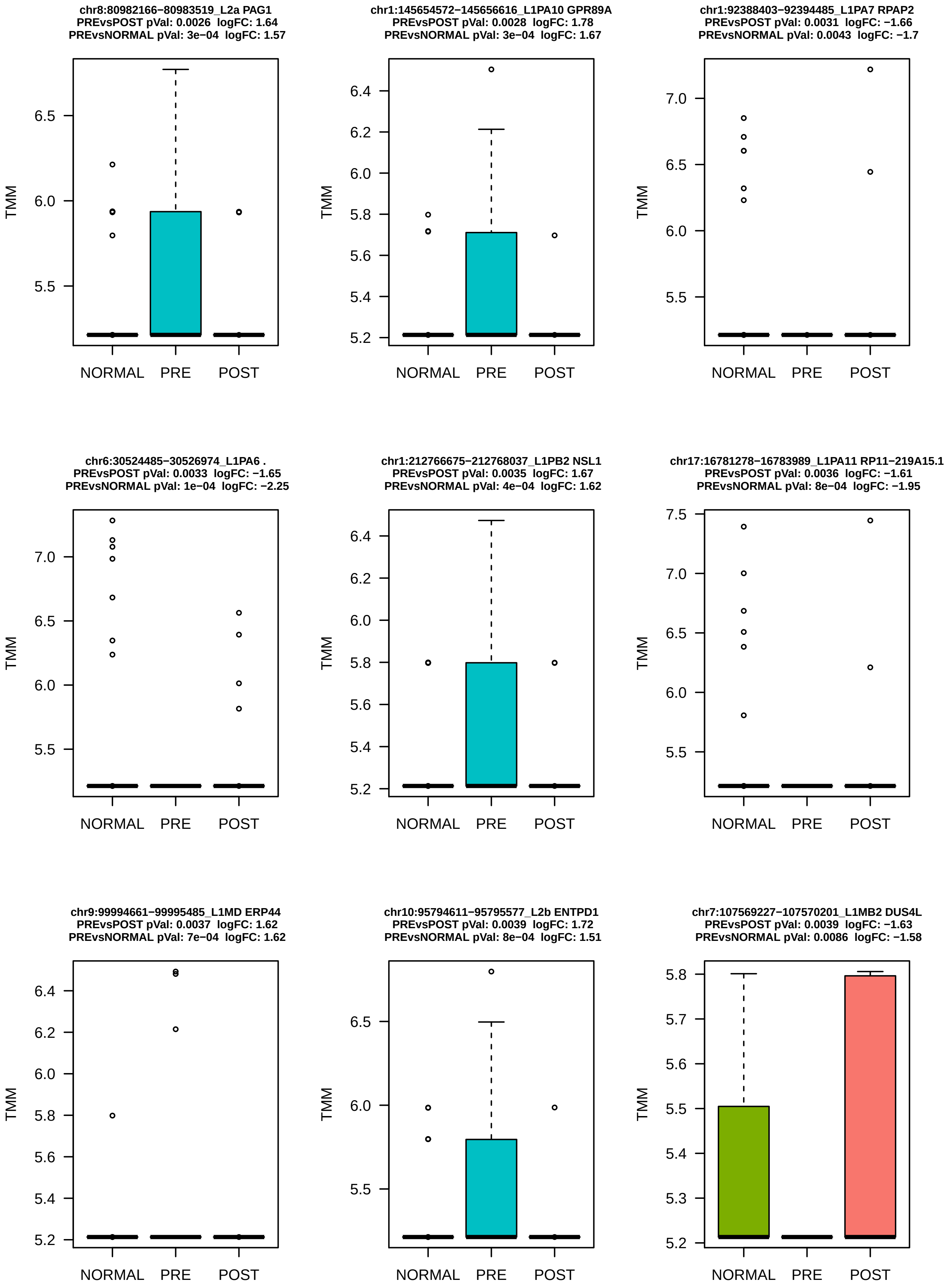

chr8:80982166−80983519_L2a PAG1
PREvsPOST pVal: 0.0026 logFC: 1.64
PREvsNORMAL pVal: 3e−04 logFC: 1.57
TMM
6.5
6.0
5.5
NORMAL
PRE
POST
chr1:145654572−145656616_L1PA10 GPR89A
PREvsPOST pVal: 0.0028 logFC: 1.78
PREvsNORMAL pVal: 3e−04 logFC: 1.67
TMM
6.4
6.2
6.0
5.8
5.6
5.4
5.2
NORMAL
PRE
POST
chr1:92388403−92394485_L1PA7 RPAP2
PREvsPOST pVal: 0.0031 logFC: −1.66
PREvsNORMAL pVal: 0.0043 logFC: −1.7
TMM
7.0
6.5
6.0
5.5
NORMAL
PRE
POST
chr6:30524485−30526974_L1PA6 .
PREvsPOST pVal: 0.0033 logFC: −1.65
PREvsNORMAL pVal: 1e−04 logFC: −2.25
TMM
7.0
6.5
6.0
5.5
NORMAL
PRE
POST
chr1:212766675−212768037_L1PB2 NSL1
PREvsPOST pVal: 0.0035 logFC: 1.67
PREvsNORMAL pVal: 4e−04 logFC: 1.62
TMM
6.4
6.2
6.0
5.8
5.6
5.4
5.2
NORMAL
PRE
POST
chr17:16781278−16783989_L1PA11 RP11−219A15.1
PREvsPOST pVal: 0.0036 logFC: −1.61
PREvsNORMAL pVal: 8e−04 logFC: −1.95
TMM
7.5
7.0
6.5
6.0
5.5
NORMAL
PRE
POST
chr9:99994661−99995485_L1MD ERP44
PREvsPOST pVal: 0.0037 logFC: 1.62
PREvsNORMAL pVal: 7e−04 logFC: 1.62
TMM
6.4
6.2
6.0
5.8
5.6
5.4
5.2
NORMAL
PRE
POST
chr10:95794611−95795577_L2b ENTPD1
PREvsPOST pVal: 0.0039 logFC: 1.72
PREvsNORMAL pVal: 8e−04 logFC: 1.51
TMM
6.5
6.0
5.5
NORMAL
PRE
POST
chr7:107569227−107570201_L1MB2 DUS4L
PREvsPOST pVal: 0.0039 logFC: −1.63
PREvsNORMAL pVal: 0.0086 logFC: −1.58
TMM
5.8
5.7
5.6
5.5
5.4
5.3
5.2
NORMAL
PRE
POST

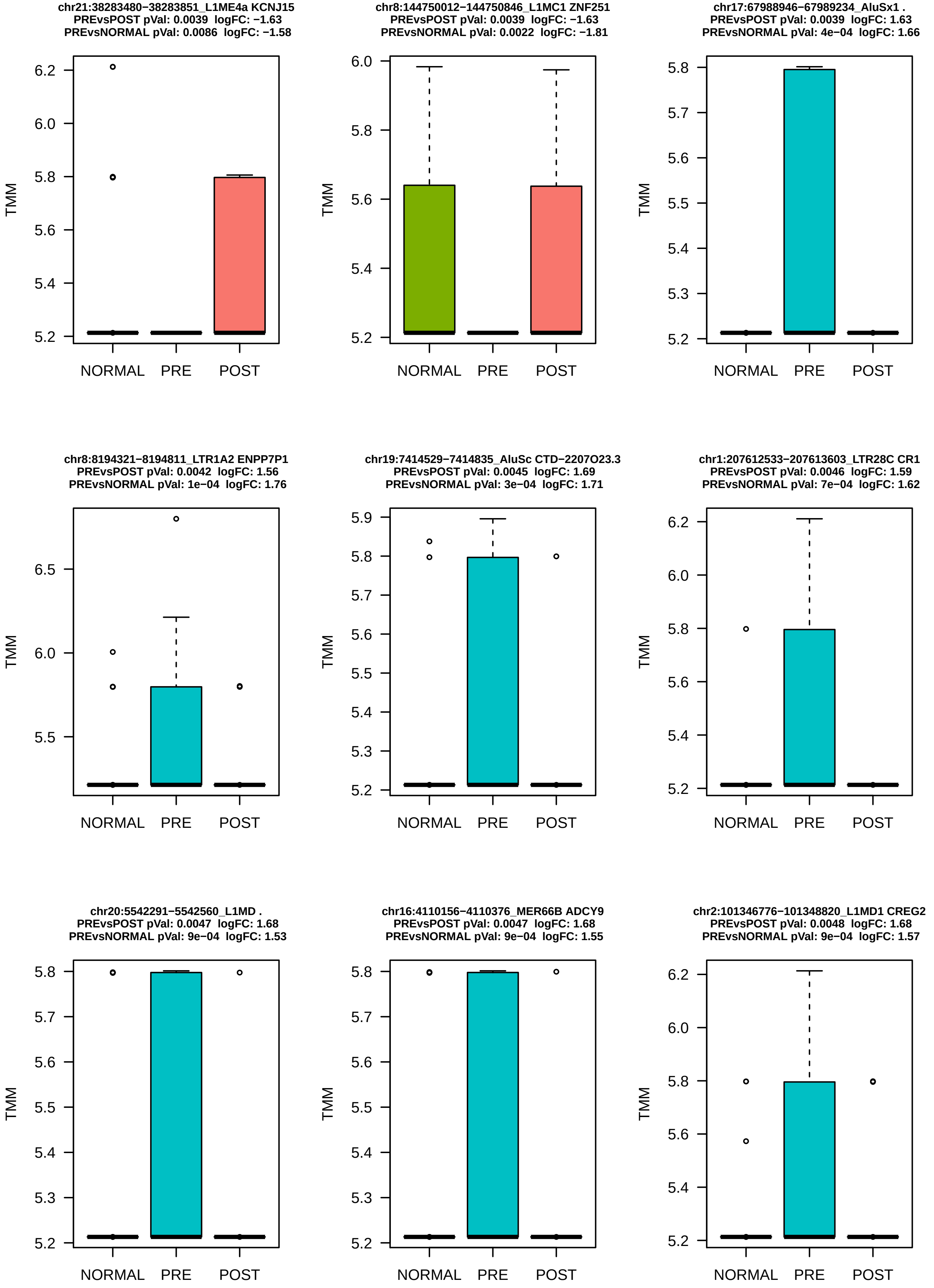
chr21:38283480−38283851_L1ME4a KCNJ15
PREvsPOST pVal: 0.0039 logFC: −1.63
PREvsNORMAL pVal: 0.0086 logFC: −1.58
chr8:144750012−144750846_L1MC1 ZNF251
PREvsPOST pVal: 0.0039 logFC: −1.63
PREvsNORMAL pVal: 0.0022 logFC: −1.81
chr17:67988946−67989234_AluSx1 .
PREvsPOST pVal: 0.0039 logFC: 1.63
PREvsNORMAL pVal: 4e−04 logFC: 1.66
chr8:8194321−8194811_LTR1A2 ENPP7P1
PREvsPOST pVal: 0.0042 logFC: 1.56
PREvsNORMAL pVal: 1e−04 logFC: 1.76
chr19:7414529−7414835_AluSc CTD−2207O23.3
PREvsPOST pVal: 0.0045 logFC: 1.69
PREvsNORMAL pVal: 3e−04 logFC: 1.71
chr1:207612533−207613603_LTR28C CR1
PREvsPOST pVal: 0.0046 logFC: 1.59
PREvsNORMAL pVal: 7e−04 logFC: 1.62
chr20:5542291−5542560_L1MD .
PREvsPOST pVal: 0.0047 logFC: 1.68
PREvsNORMAL pVal: 9e−04 logFC: 1.53
chr16:4110156−4110376_MER66B ADCY9
PREvsPOST pVal: 0.0047 logFC: 1.68
PREvsNORMAL pVal: 9e−04 logFC: 1.55
chr2:101346776−101348820_L1MD1 CREG2
PREvsPOST pVal: 0.0048 logFC: 1.68
PREvsNORMAL pVal: 9e−04 logFC: 1.57
TMM
NORMAL
PRE
POST

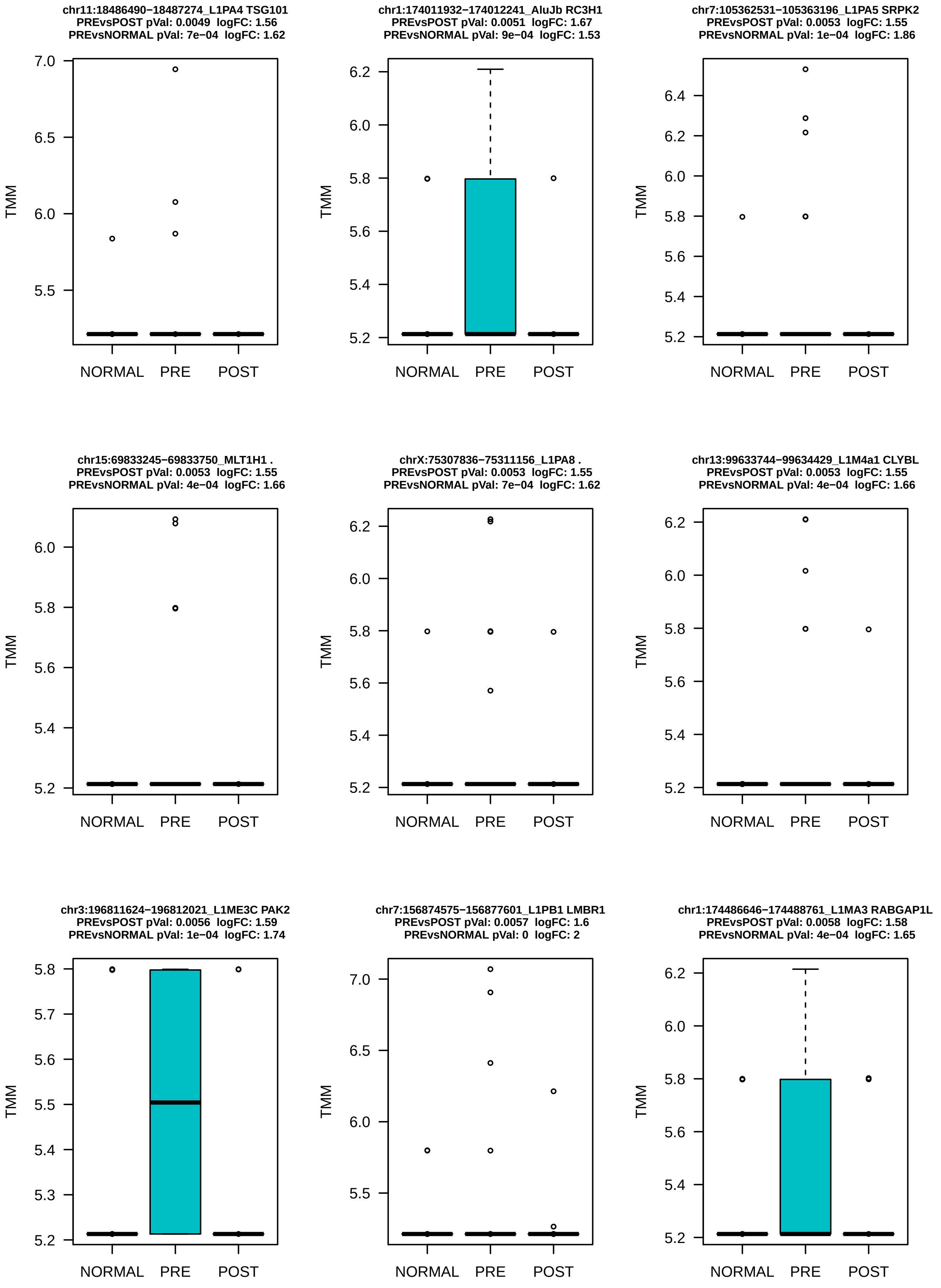
chr11:18486490−18487274_L1PA4 TSG101
PREvsPOST pVal: 0.0049 logFC: 1.56
PREvsNORMAL pVal: 7e−04 logFC: 1.62
chr1:174011932−174012241_AluJb RC3H1
PREvsPOST pVal: 0.0051 logFC: 1.67
PREvsNORMAL pVal: 9e−04 logFC: 1.53
chr7:105362531−105363196_L1PA5 SRPK2
PREvsPOST pVal: 0.0053 logFC: 1.55
PREvsNORMAL pVal: 1e−04 logFC: 1.86
chr15:69833245−69833750_MLT1H1 .
PREvsPOST pVal: 0.0053 logFC: 1.55
PREvsNORMAL pVal: 4e−04 logFC: 1.66
chrX:75307836−75311156_L1PA8 .
PREvsPOST pVal: 0.0053 logFC: 1.55
PREvsNORMAL pVal: 7e−04 logFC: 1.62
chr13:99633744−99634429_L1M4a1 CLYBL
PREvsPOST pVal: 0.0053 logFC: 1.55
PREvsNORMAL pVal: 4e−04 logFC: 1.66
chr3:196811624−196812021_L1ME3C PAK2
PREvsPOST pVal: 0.0056 logFC: 1.59
PREvsNORMAL pVal: 1e−04 logFC: 1.74
chr7:156874575−156877601_L1PB1 LMBR1
PREvsPOST pVal: 0.0057 logFC: 1.6
PREvsNORMAL pVal: 0 logFC: 2
chr1:174486646−174488761_L1MA3 RABGAP1L
PREvsPOST pVal: 0.0058 logFC: 1.58
PREvsNORMAL pVal: 4e−04 logFC: 1.65
TMM
NORMAL
PRE
POST

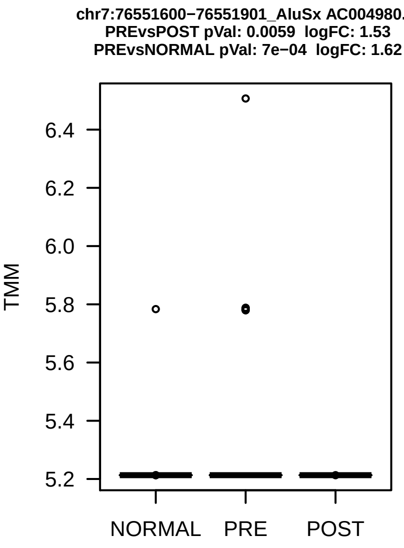
chr7:76551600−76551901_AluSx AC004980.7
PREvsPOST pVal: 0.0059 logFC: 1.53
PREvsNORMAL pVal: 7e−04 logFC: 1.62
TMM
6.4
6.2
6.0
5.8
5.6
5.4
5.2
NORMAL
PRE
POST

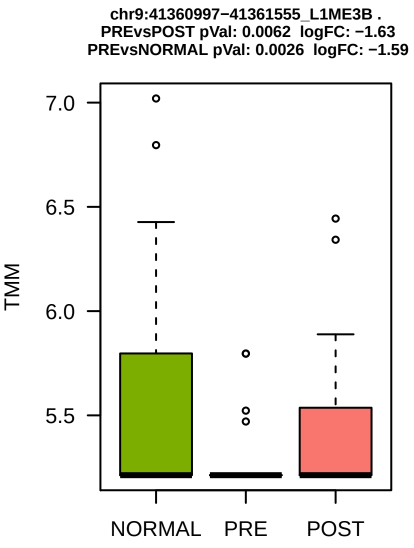
chr9:41360997−41361555_L1ME3B .
PREvsPOST pVal: 0.0062 logFC: −1.63
PREvsNORMAL pVal: 0.0026 logFC: −1.59
TMM
7.0
6.5
6.0
5.5
NORMAL
PRE
POST

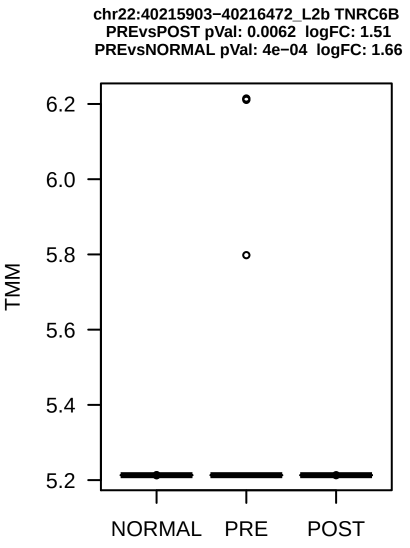
chr22:40215903−40216472_L2b TNRC6B
PREvsPOST pVal: 0.0062 logFC: 1.51
PREvsNORMAL pVal: 4e−04 logFC: 1.66
TMM
6.2
6.0
5.8
5.6
5.4
5.2
NORMAL
PRE
POST

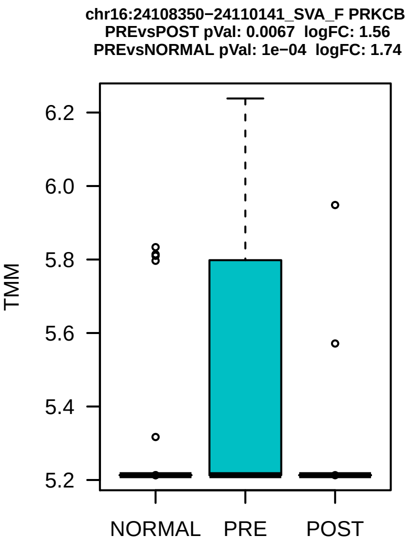
chr16:24108350−24110141_SVA_F PRKCB
PREvsPOST pVal: 0.0067 logFC: 1.56
PREvsNORMAL pVal: 1e−04 logFC: 1.74
TMM
6.2
6.0
5.8
5.6
5.4
5.2
NORMAL
PRE
POST

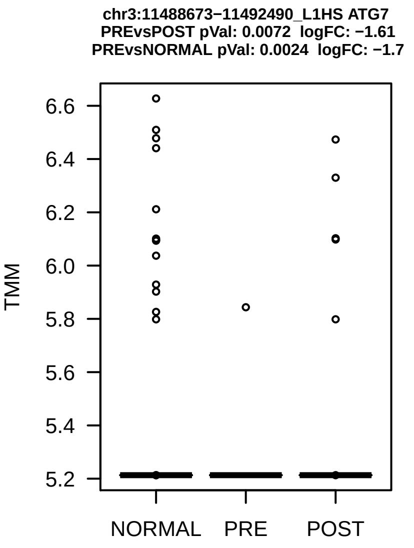
chr3:11488673−11492490_L1HS ATG7
PREvsPOST pVal: 0.0072 logFC: −1.61
PREvsNORMAL pVal: 0.0024 logFC: −1.7
TMM
6.6
6.4
6.2
6.0
5.8
5.6
5.4
5.2
NORMAL
PRE
POST

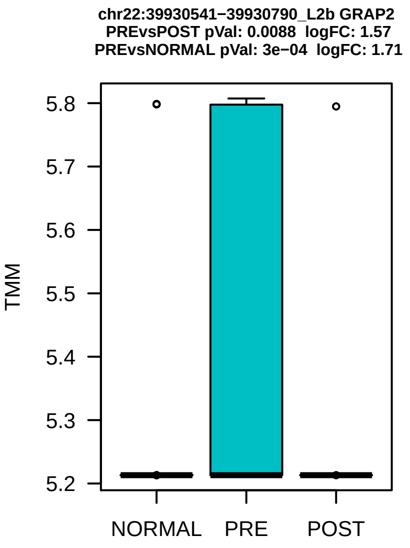
chr22:39930541−39930790_L2b GRAP2
PREvsPOST pVal: 0.0088 logFC: 1.57
PREvsNORMAL pVal: 3e−04 logFC: 1.71
TMM
5.8
5.7
5.6
5.5
5.4
5.3
5.2
NORMAL
PRE
POST

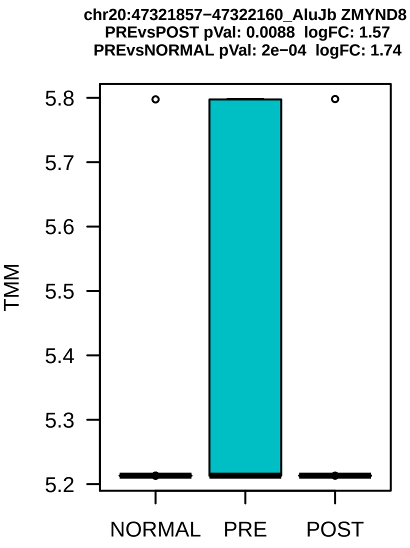
chr20:47321857−47322160_AluJb ZMYND8
PREvsPOST pVal: 0.0088 logFC: 1.57
PREvsNORMAL pVal: 2e−04 logFC: 1.74
TMM
5.8
5.7
5.6
5.5
5.4
5.3
5.2
NORMAL
PRE
POST

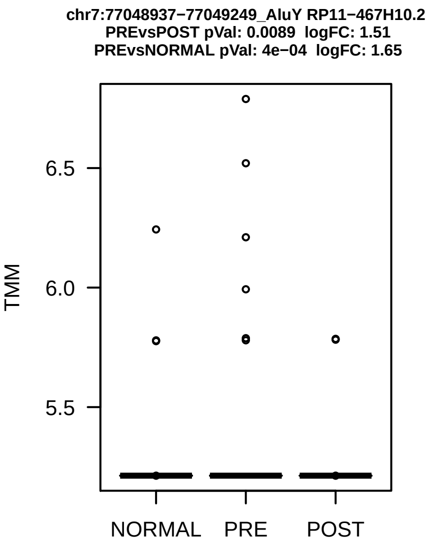
chr7:77048937−77049249_AluY RP11−467H10.2
PREvsPOST pVal: 0.0089 logFC: 1.51
PREvsNORMAL pVal: 4e−04 logFC: 1.65
TMM
6.5
6.0
5.5
NORMAL
PRE
POST
