# Supplementary figures and images for: A retrotransposon storm marks clinical phenoconversion to late-onset Alzheimer’s disease
Source: GeroScience. 2022 May 19;44(3):1525–50. doi: 10.1007/s11357-022-00580-w (PMC9213607; doi:10.1007/s11357-022-00580-w)

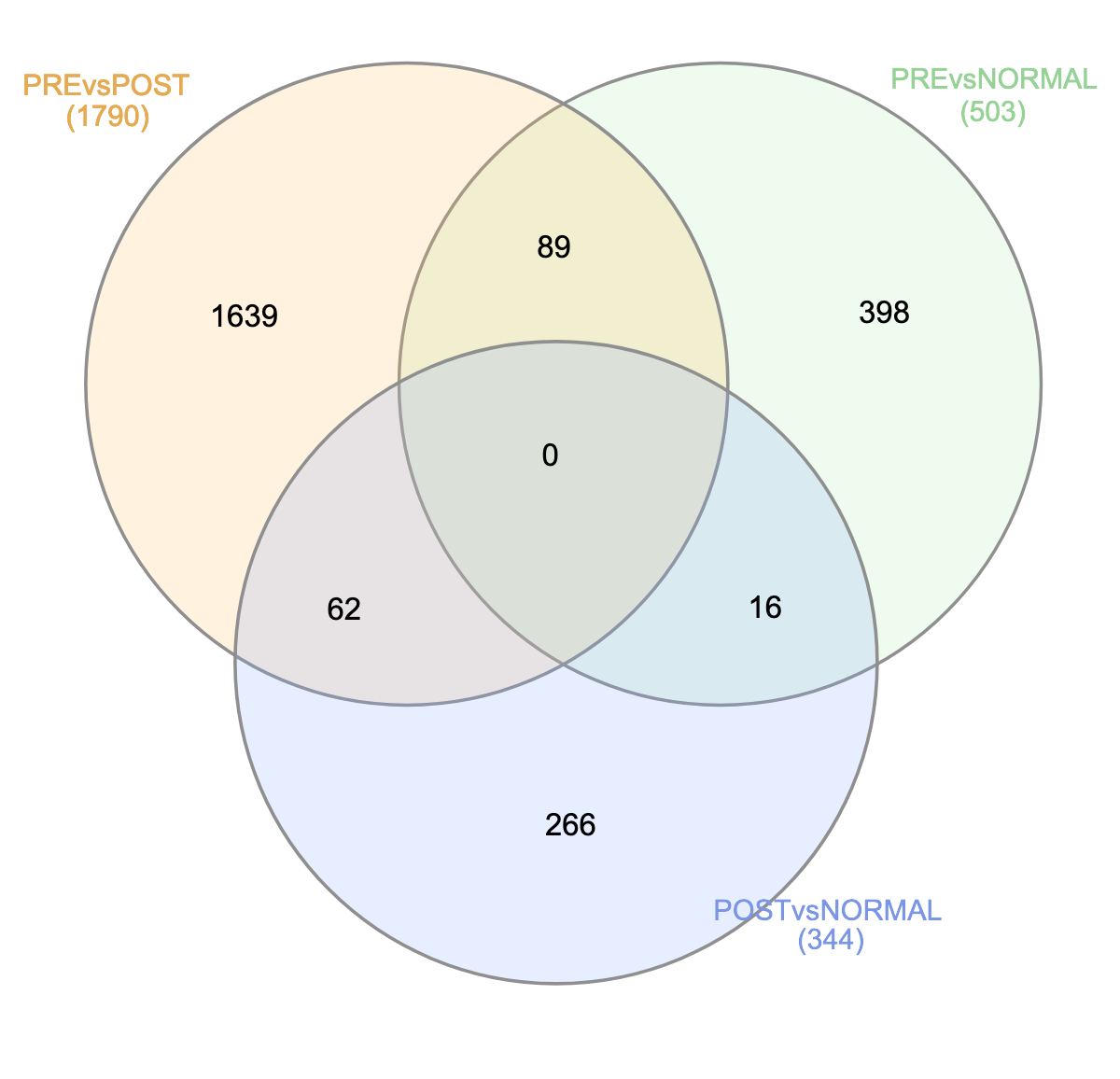

Supplement: Supplementary file 3 — Supplementary file3 (PNG 154 KB) [file 11357_2022_580_MOESM3_ESM.png]
